# Polariton transport in 2D semiconductors: Phonon-mediated transitions between ballistic, superdiffusive, and exciton-limited regimes

**DOI:** 10.1126/sciadv.aea3495

**Published:** 2025-11-14

**Authors:** Jamie M. Fitzgerald, Roberto Rosati, Ermin Malic

**Affiliations:** Department of Physics, Philipps-Universität Marburg, 35032 Marburg, Germany.

## Abstract

Exciton transport in 2D semiconductors holds promise for room temperature, ultracompact optoelectronic devices, but it is limited by short propagation distances. Hybridization of excitons with cavity photons to form exciton-polaritons can enhance the propagation by orders of magnitude, enabling a coherent, ballistic transport. However, a microscopic understanding of the role of phonons is still lacking, particularly regarding their influence on the crossover from the ballistic to the diffusive polariton transport regime. Here, we investigate the spatiotemporal polariton dynamics in MoSe_2_ monolayers at moderate to high temperatures, explicitly including the phonon-mediated coupling to the intervalley exciton reservoir. We identify three distinct transport regimes: (i) an initial subpicosecond ballistic-like regime characterized by a phonon-induced velocity renormalization, (ii) a transient, few-picosecond superdiffusive regime characterized by strongly enhanced diffusion, and (iii) a slower, exciton-limited diffusion following thermalization. The gained microscopic insights will trigger and guide future experimental studies on the phonon-mediated polariton transport in atomically thin semiconductors.

## Teaser

Scattering with phonons dictates the crossover from ballistic to diffusive exciton polariton transport in 2D semiconductors.

## INTRODUCTION

Monolayers of transition metal dichalcogenides (TMDs) support tightly bound Wannier excitons that are stable at room temperature and interact strongly with light (*1*–*3*). Given their charge neutrality, two-dimensional nature, and quasi-bosonic statistics, there has been long-standing interest in the in-plane transport properties of TMD excitons for both fundamental many-body physics and technological applications (*4*, *5*). In particular, exciton transport plays a key role in light-energy conversion, including photovoltaics (*6*), light-emitting diodes (*7*), and photosynthesis (*8*). There is also great potential for fast and energy-efficient excitonic circuits that bridge nano-scale electronics and micrometer-scale photonics (*9*).

At elevated temperatures and low excitation power, a spatially localized population of excitons spreads isotropically via conventional phonon-driven diffusion, as described by Fick’s law (*4*). The resulting photoluminescence (PL) from exciton recombination can be used to track exciton density in both time and space (*10*–*14*), while dielectric (*15*, *16*) and strain engineering (*17*, *18*) have been demonstrated to direct the flow of exciton current. The rich exciton landscape of TMDs (*19*) is fundamental to understanding exciton diffusion, since the relative position of bright and dark exciton states is sensitive to the dielectric environment (*20*) and strain (*21*, *22*), as demonstrated by exciton antifunnelling observed in tungsten-based TMDs (*23*). Furthermore, vertical stacking of heterostructures gives rise to spatially separated and long-lived interlayer excitons with permanent out-of-plane dipole moments (*24*). This allows for electric field (*9*, *25*) and twist-angle (*26*) control of exciton diffusion. Despite these notable attributes, exciton transport is inherently slow, with typical diffusion coefficients on the order of 1 to 10 cm^2^/s in TMD monolayers (*12*).

In contrast, photons are ideal information and energy carriers that can propagate over macroscopic length scales in materials at a substantial fraction of the speed of light (*27*). However, developing compact integrated photonic circuitry necessitates strong nonlinearities at low optical intensities (*28*). Exciton-polaritons are hybrid light-matter states that promise the best of both worlds, inheriting a light effective mass from their photon constituent, and tunability and nonlinearity from their material component (*29*). This allows them to be manipulated using electric (*30*) and magnetic (*31*, *32*) fields. They are also more resistant to disorder than pure excitons due to the motional narrowing effect (*33*). Over the past decades, exciton polariton transport has been explored in conventional semiconductors in both microcavity (*34*, *35*) and waveguide structures (*36*). More recently, there has been intense focus on materials with a large Rabi splitting that can support long-range polariton propagation at room temperature, such as organics (*37*–*41*), perovskites (*42*–*45*), and TMDs (*33*, *46*–*49*). In particular, recent pioneering experiments have reported a detuning-dependent crossover between ballistic and diffusive regimes in exciton polariton transport (*40*, *41*). Yet, key questions remain regarding the microscopic origins of this crossover, namely, how ballistic, coherent transport can persist despite nonresonant excitation and strong phonon interactions, and what are the characteristics of the system’s evolution from the ballistic to the diffusive regime on a few-picosecond timescale. A crossover from ballistic to diffusive polariton transport has also been predicted to occur between the limits of weak and strong static excitonic disorder (*50*). In the context of TMDs, the ballistic propagation of polaritons across tens of micrometers has been demonstrated, along with trapping and manipulation using a spatially dependent modification of the cavity length (*33*). However, the role that dark intervalley excitons play in polariton transport is still largely unknown. In our previous work, the full exciton landscape was shown to be essential for understanding polariton optics (*51*, *52*) and relaxation dynamics (*53*). For the latter, intervalley KK′ excitons were shown to act as additional exciton reservoir to efficiently populate polariton states and bypass the relaxation bottleneck (*54*).

**Fig. 1.**
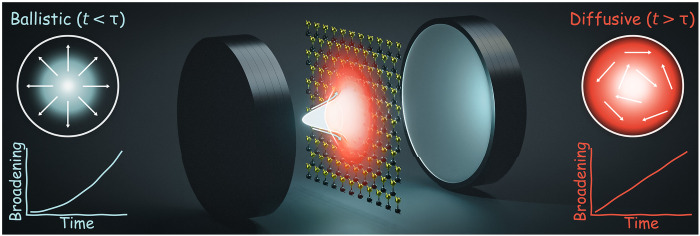
Schematic of ballistic and diffusive transport regimes. Illustration of a MoSe_2_ monolayer integrated within a Fabry-Pérot microcavity. The system is in the strong coupling regime, where transport behavior is heavily modified because of the large group velocities of exciton-polaritons. The ballistic (left, blue) and diffusive (right, red) regimes are depicted, with the corresponding behavior of the real-space broadening in time. Here, τ represents a typical thermalization time of the system and dictates the crossover from rapid ballistic-like expansion (quadratic) to a slower diffusive regime (linear).

In this work, we focus on a representative hBN-encapsulated MoSe_2_ monolayer integrated within a Fabry-Pérot microcavity (Fig. 1) and calculate the full spatiotemporal dynamics of exciton-polaritons by solving the polariton Boltzmann transport equation. Our model operates in the linear (low-excitation) regime, fully accounting for polariton-phonon scattering while neglecting nonlinear polariton-polariton interactions [e.g., pump-induced changes of the polariton potential (*55*)]. Crucially, we include the momentum-resolved, real-space dynamics of both polaritons and dark intra- and intervalley reservoir excitons. This provides a microscopic description of the phonon-driven spatiotemporal relaxation of the system, which is explored for different temperatures and detunings. We observe three distinct regimes in the time evolution of the polariton cloud: First, there is a rapid ballistic-like expansion driven by the ultrafast relaxation of hot excitons into polaritons. This is followed by a transient superdiffusive regime where the expansion slows down over time, but is still up to three orders of magnitude faster than regular exciton diffusion. This occurs due to phonon-driven scattering between the polariton states within the lightcone and the exciton reservoir. Last, in the steady-state limit, the system settles into a much slower, exciton-limited diffusive regime. However, the propagation is still enhanced relative to the conventional bare exciton case by the presence of a cavity. In addition, we establish that polariton transport is strongly dependent on key experimental parameters. Specifically, temperature, cavity detuning, and nonresonant excitation conditions act as effective levers to manipulate the transport dynamics of both polaritons and reservoir excitons, providing a framework for future experiments to probe the fundamental role of phonons. Overall, our work provides a material-realistic and predictive theoretical foundation for phonon-mediated polariton transport under technologically relevant conditions.

## RESULTS

**Fig. 2.**
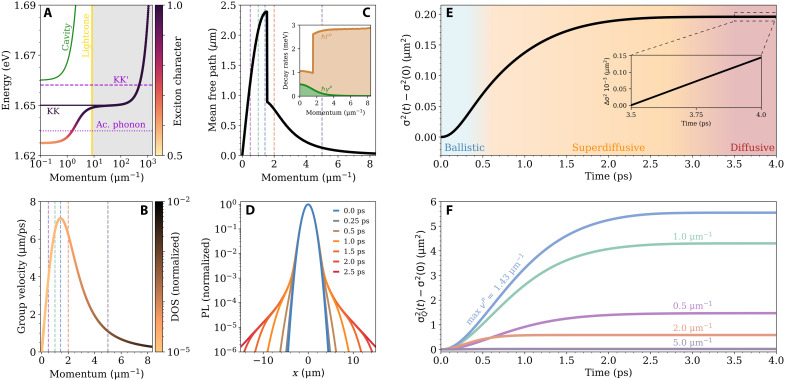
Ballistic, superdiffusive and diffusive transport regimes. (**A**) Lower polariton energy for a MoSe_2_ monolayer integrated within a microcavity that is detuned by +10 meV. The colormap shows the excitonic character, which is 62% at *Q* = 0. The dashed purple line denotes the position of dark KK′ excitons, while the dotted line shows the energy at which intervalley scattering processes into KK′ exciton states are possible via zone-edge acoustic phonons. (**B**) The corresponding group velocity of the lower polariton, with the density of states indicated by the colormap (log scale). (**C**) The momentum-resolved polariton mean free path with the polariton radiative decay rate (green) and the phonon-induced scattering rate (brown) shown in the inset. (**D**) Time evolution of the normalized momentum-integrated polariton photoluminescence profile (log scale) showing a non-Gaussian expansion. (**E**) The corresponding broadening of the momentum-integrated polariton density, illustrating a crossover from an ultrafast ballistic-like regime in the first few hundred femtoseconds (blue shading), to a transient superdiffusive regime from ~0.5 → 3 ps (orange shading), and lastly to a much slower exciton-like diffusion (red shading). The latter regime is characterized by a linear slope, as shown in the inset. (**F**) The momentum-resolved broadening at five representative momenta (corresponding to the dashed vertical lines in B and C).

We consider an hBN-encapsulated MoSe_2_ monolayer integrated within a λ/2 Fabry-Pérot microcavity that is blue-detuned by 10 meV at zero in-plane momentum. By solving the Wannier equation (*56*), monolayer MoSe_2_ is found to be a direct semiconductor, where the momentum-dark KK′ excitons (dashed purple line in Fig. 1A) lie about 10 meV (*57*) above the bottom of the bright KK exciton at 1.65 eV (*58*). To account for the coupling between excitons and the cavity mode, a Hopfield transformation is used (*53*, *59*). The calculated Rabi splitting is 39 meV, and the lower polariton has an excitonic character of 62% at *Q* = 0 (Fig. 2A). Further details on the Wannier-Hopfield model can be found in Methods.

In stark contrast to excitons, a key feature of polaritons is their momentum-dependent photonic/excitonic character and sharp dispersion within the lightcone. Focusing on the lower polariton branch, this has two important implications for transport: First, it leads to large group velocities, $v_{Q}^{P}$, on the order of 1 to 10 μm/ps, which peak at the inflection point of the dispersion (Fig. 2B). These velocities are ~10^4^ larger than those of typical excitons in the lightcone (<1 nm/ps). This in turn implies very small effective masses (*59*) and density of states (DOS) (color gradient in Fig. 2B). Second, it leads to a sharp momentum dependence of the polariton-phonon scattering rate within the lightcone, arising from the opening of specific phonon scattering channels into both momentum-direct (i.e., KK) and momentum-indirect excitons (e.g., KK′) (*51*–*53*, *60*). A consequence of this is a nontrivial momentum dependence of the polariton mean free path, $L_{Q}=v_{Q}^{P}\tau_{Q}$, as shown in Fig. 2C at 300 K. At the momentum of 1.55 μm^−1^, there is a sharp drop in the mean free path, corresponding to the opening of the acoustic phonon scattering channel into the KK′ exciton reservoir (purple dotted line in Fig. 2A). Below this momentum the phonon energy of 18 meV (*61*) is not sufficient to scatter a polariton into a KK′ exciton state (*53*). These zone-edge phonons are responsible for the ultrafast valley depolarization of carriers observed in MoSe_2_ monolayers (*62*). When this scattering channel opens up, there is a sharp increase in the phonon-induced polariton scattering rate (filled brown area in the inset of Fig. 2C), $\Gamma_{Q}$, and a corresponding drop in the polariton decay time, $\tau_{Q}=1/\left[2\left(\gamma_{Q}+\Gamma_{Q}\right)\right]$. Note that at all momenta, both the intravalley KK and intervalley optical channels are open due to the higher phonon energies of 34 and 33 meV, respectively (*61*). This means that lower polaritons can be populated and there is no bottleneck (*53*). The radiative decay rate of the lower polariton, $\gamma_{Q}$, provides a sizeable contribution to the overall decay rate at low momenta within the lightcone (filled green area in the inset of Fig. 2C). Note that the possible impact of leaky modes at higher angles in the lightcone has been neglected (*54*).

### Distinct polariton transport regimes

We solve the Boltzmann transport equation to provide a microscopic description of the spatiotemporal dynamics of the exciton polariton Wigner function (*63*, *64*), $N_{nQ}\left(R\right)$, which gives the spatially dependent quasi-distribution of excitons/polaritons with center-of-mass momentum Q and valley/branch *n*. A key theoretical advance of our model is the inclusion of both exciton/polariton-phonon scattering and the full spatiotemporal dynamics of the exciton reservoir states in the KK and KK′ valleys. Our study specifically addresses the low-excitation regime, allowing us to unambiguously clarify the role of exciton/polariton-phonon scattering. Furthermore, we consider a nonresonant excitation with an initial hot-exciton population in the KK reservoir, i.e., $N_{nQ}\left(R,t\right)$ describes an incoherent polariton population. The initial spatial distribution of the exciton polariton cloud corresponds to a Gaussian with a full width at half maximum (FWHM) of 2 μm. Further details can be found in Methods and the Supplementary Materials.

We first focus on the spatiotemporal dynamics of polariton states within the lightcone at room temperature. Figure 2C shows the time evolution of a slice of the normalized momentum-integrated polariton PL spatial profile (i.e., integrated over all momenta within the lightcone) on a logarithmic scale. A non-Gaussian expansion of the polariton cloud is immediately apparent, with rapid micrometer-scale broadening occurring in the tail region over about 3 ps, while the central region of high intensity remains largely unchanged. This is in contrast to bare exciton diffusion, which is typically well modeled by a Gaussian with a time-dependent variance in rotationally invariant systems (*12*). This difference can be attributed to a small population of fast polaritons in the lightcone and a vastly larger population of slow reservoir excitons, which are effectively static on these timescales. As the rapid polaritons radiatively decay or scatter with phonons, the static reservoir is continuously repopulating the polariton occupation, keeping the bulk of the polariton cloud fixed in place. This non–shape-preserving expansion of exciton polaritons has been observed experimentally in TMD (*33*), perovskite (*43*), and amorphous molecular systems (*39*).

Figure 2E illustrates the time-dependent broadening of the integrated polariton cloud shown in Fig. 2D. We plot the time-dependent contribution of the squared width, i.e., $\sigma^{2}\left(t\right)-\sigma^{2}\left(0\right)$, where $\sigma^{2}\left(t\right)=\int N\left(R,t\right)R^{2}d^{2}R/\;\int N\left(R,t\right)d^{2}R$, N(R,t) is the spatial density obtained by integrating the Wigner function over momentum, and $\sigma^{2}\left(0\right)$ is determined by the initial spatial profile. This is directly extractable from time- and spatially resolved PL (*12*) and pump-probe (*41*) measurements commonly used in transport studies of excitons and polaritons. Three different regimes of transport are immediately apparent. Below 300 fs, there is a fast ballistic-like expansion that is characterized by a quadratic time dependence (*65*) (blue region). In this regime, the polariton occupation grows quickly as it is fed from the nonthermalized KK and KK′ exciton reservoirs (see fig. S5). Consequently, the polariton cloud expands rapidly as any losses are compensated by the efficient in-scattering. As the growth in the polariton occupation saturates and the system approaches a characteristic timescale where the majority of polaritons have scattered with a phonon or radiatively decayed, the rate of expansion drops and the system smoothly evolves into a transient superdiffusive regime (*66*, *67*) (orange region). Here, the time dependence of the broadening is no longer quadratic but the polariton cloud still expands at a much larger rate than bare excitons. Last, as the system tends toward the steady state, the rate of expansion decreases by orders of magnitude and appears flat on the scale of Fig. 1E (red region). Closer inspection reveals a linear slope (see inset), which is characteristic of the conventional diffusive regime (*65*). Here, the rate of expansion is about three orders of magnitude smaller than in the earlier transient ballistic-like and superdiffusive regimes. The associated diffusion coefficient corresponds to typical excitonic values of around 1 cm^2^/s in MoSe_2_ monolayers (*14*, *68*). This reveals that the polariton subsystem has reached thermal equilibrium with the exciton reservoir due to the strong scattering with phonons at room temperature. Overall, the time evolution of the polariton broadening shows that ballistic-like propagation of polaritons is possible at room temperature, even for polaritons with a substantial excitonic component and in the presence of strong scattering with phonons. However, this is a short-lived subpicosecond behavior, after which the exciton polariton cloud expands in a cavity-enhanced diffusive fashion. This is in excellent qualitative agreement with the time-resolved expansion dynamics of the polariton cloud observed for organic semiconductors (*40*), which also revealed three distinct transport regimes on similar timescales for polaritons of intermediate exciton/photonic character.

Further insight is provided in Fig. 2F, where the momentum-resolved broadening is shown for five illustrative momenta indicated by the dashed verticals lines in Fig. 2 (B and C). Because of the one-to-one correspondence between momentum and emission angle, the momentum space distribution of polaritons in the lightcone can be tracked in real space via PL spectroscopy (*35*). Considering the first three momenta (purple, green, and blue lines), both the expansion rate and extent of the polariton expansion show a clear dependence on the corresponding group velocity, i.e., a larger $v_{Q}^{P}$ leads to a greater broadening. The peak group velocity of 7.1 μm/ps at 1.43 μm^−1^ corresponds to the polariton cloud component that expands most rapidly and furthest (blue line), with $\sigma^{2}-\sigma {\left(0\right)}^{2}$ reaching 5.3 μm^2^ within 2 ps. For *Q* = 2 μm^−1^ (peach curve), we observe a drastic drop in the broadening, despite the large, near-maximum group velocity at this momentum. This is a consequence of the opening of the acoustic phonon scattering channel to KK′ excitons at 1.55 μm^−1^ (Fig. 2C). This means an increased scattering rate; hence, polaritons with these momenta exhibit a shorter ballistic-like and superdiffusive regime, leading to a reduced real-space expansion. Last, we find for larger momenta, such as 5 μm^−1^ (lavender curve), a very small broadening that is barely visible on the scale of Fig. 2F. These much slower polaritons possess a very large DOS relative to the more rapid polaritons at lower momenta (Fig. 2B); hence, their contribution to the integrated polariton density in Fig. 2E is dominant. This explains the significantly reduced broadening (~25 times smaller) of the momentum-integrated polariton cloud in Fig. 2E compared to the momentum-resolved results for the most rapid polaritons shown in Fig. 2F. This material-specific prediction is directly testable using techniques such as momentum-resolved ultrafast polariton imaging (*41*). Furthermore, the underlying many-particle mechanism, namely, the opening of intervalley scattering pathways, has recently been experimentally observed in polariton detuning-dependent reflection spectra for monolayer WSe_2_ (*52*).

**Fig. 3.**
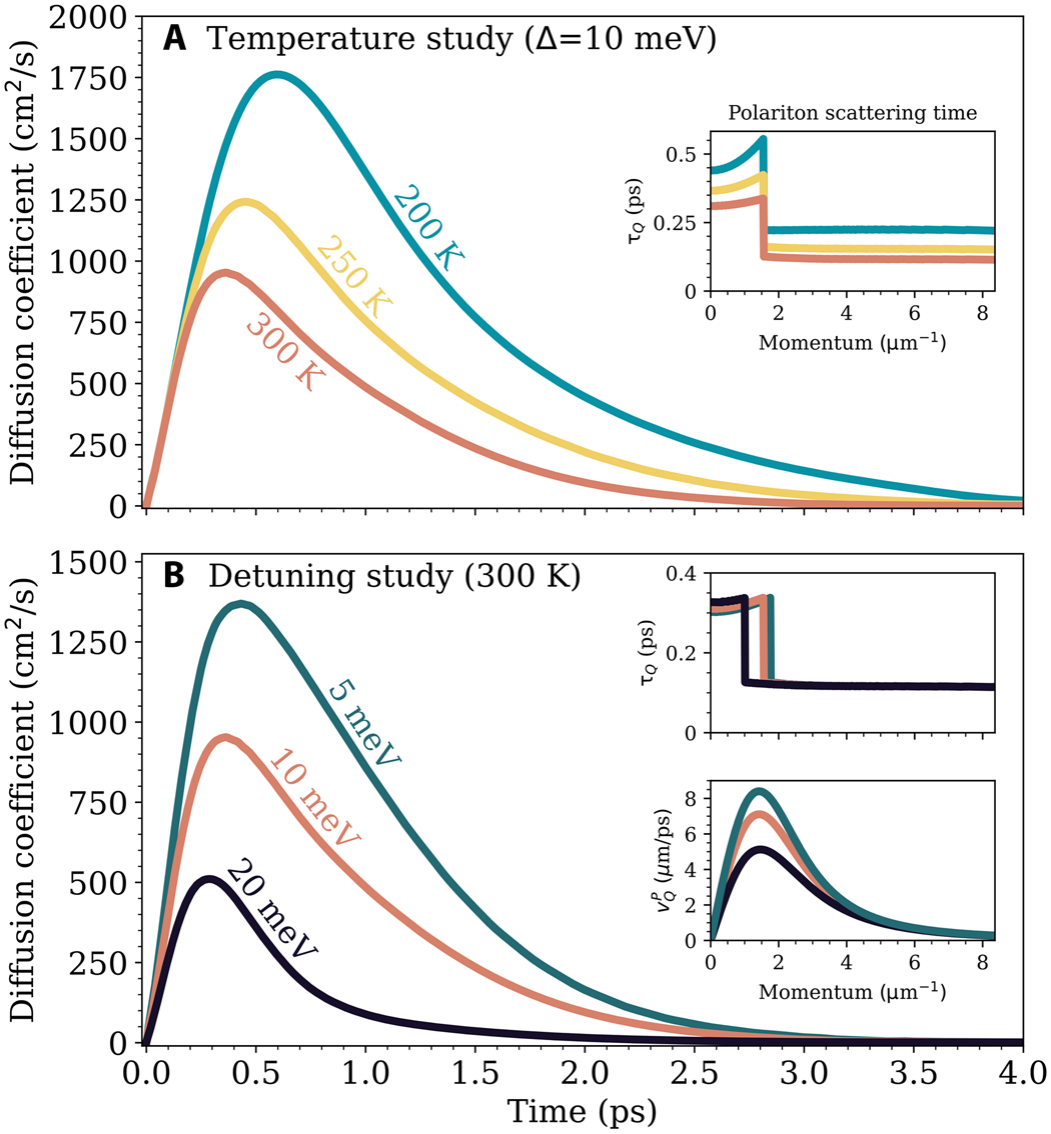
Temperature and detuning dependence of polariton transport. (**A**) Temporal evolution of the temperature-dependent polariton diffusion coefficient at a fixed detuning Δ = +10 meV. The inset shows the corresponding momentum-resolved polariton scattering time, $\tau_{Q}$. (**B**) Detuning study of the polariton diffusion coefficient at room temperature. The insets show the momentum-dependent polariton scattering time (top) and the group velocity (bottom).

To further elucidate the different transport regimes, Fig. 3A shows the effective diffusion coefficient (*64*, *66*), $D\left(t\right)=\partial_{t}\sigma^{2}\left(t\right)/4$, of the momentum-integrated polariton cloud for three different temperatures (momentum-resolved results are shown in fig. S3). Focusing first on room temperature (red curve), there is an initial linear increase of the diffusion coefficient with time, indicating a ballistic-like regime (*64*). After initialization of a hot-exciton population, there is a swift energy relaxation via efficient phonon emission from both the KK and KK′ exciton reservoir (*53*). This leads to a rapid occupation of polariton states within the lightcone over a 100 to 300 fs timescale (see fig. S5) and means that any scattering with phonons or radiative decay in that time period is compensated by the feeding of polaritons from the exciton reservoirs. In a sense, the efficient phonon-driven scattering into polariton states mimics a driving term, and hence leads to a ballistic-like transport. Next, the diffusion coefficient peaks at a large magnitude of 954 cm^2^/s at 0.36 ps, about three orders of magnitude larger than typical exciton diffusion coefficients (*14*, *68*). Over the next 2 to 3 ps, the diffusion decreases, but still remains significantly enhanced compared to the cavity-free case. For example, at 2 ps, the diffusion coefficient is still about 10 times larger than conventional exciton values. This intermediate superdiffusive regime corresponds to a nonequilibrium state, where the ballistic-like propagation has been terminated by polariton-phonon scattering into the exciton reservoirs, which acts as a frictional mechanism. Eventually, the diffusion limits to a small constant value, indicating the crossover into regular exciton-limited diffusion at steady state described by Fick’s law (*64*). Our results are consistent with reports of a dynamic crossover from a ballistic to diffusive regime for polaritons with a predominantly excitonic character in molecular materials (*40*). Similar transient superdiffusive transport has also been observed for highly hot quasi-free carrier dominated expansion in TMDs (*68*), hybrid perovskites (*69*), and silicon (*66*). Our work reveals a fundamentally different physical origin. In addition to mechanisms based on the excess kinetic energy of hot carriers, we establish that superdiffusive transport can be driven by an efficient in-scattering from the exciton reservoir compensating for phonon-induced scattering. This allows the polariton cloud to expand at an appreciable fraction of its inherent group velocity, representing a previously unidentified physical origin for superdiffusive transport in polaritonic systems.

We find a very similar ballistic-like evolution for all three temperatures up to times of about 300 to 500 fs (Fig. 3A). This is because the in-scattering mechanism is due to phonon emission, which is only weakly sensitive to temperature. We also observe both an increased maximum diffusion coefficient (1243 and 1762 cm^2^/s for 250 and 200 K, respectively) and that it takes a longer time to reach this peak as temperature decreases (0.45 and 0.59 ps for 250 and 200 K, respectively). This is due to an enhanced and extended superdiffusive regime at lower temperatures, which is caused by a longer polariton-phonon scattering time within the lightcone (see the inset). For example, at 3 ps, the diffusion coefficient for the system at 200 K is about 24 times larger than the corresponding value at 300 K. This is in contrast to the weak temperature dependence of the bare exciton diffusion coefficient in MoSe_2_ monolayers (see fig. S8).

A key advantage of polaritons is the external control of their dispersion via cavity detuning, Δ. In the context of transport, this is especially valuable as it allows for control over both the group velocity and available polariton-phonon scattering channels (*51*–*53*). Figure 3B shows the detuning dependence of the diffusion coefficient at 300 K. For smaller detuning, we find a larger peak diffusion coefficient, reaching 1370 cm^2^/s for Δ = 5 meV. This value is about 2.7 times larger than that observed for Δ = 20 meV. This enhanced superdiffusivity is a consequence of the larger group velocity (bottom inset), which grows with decreasing detuning as polaritons gain photonic character. In contrast to the group velocity, the scattering rate is relatively unaffected by detuning, and only the momentum of the opening of the acoustic phonon channel slightly shifts (top inset). As a result, there is only a small dependence of the peak polariton diffusion on detuning (0.29 and 0.42 ps for +20 and +5 meV detuning, respectively). However, the less-detuned cavity shows a prolonged superdiffusivity. For example, at 3 ps the Δ = 5 meV system exhibits a diffusion coefficient about seven times larger than the Δ = 20 meV cavity. These results illustrate that the crossover from ballistic-like to conventional diffusive regime is sensitive to the excitonic/photonic character of polaritons, in good agreement with experimental observations across different material systems (*40*, *41*). As the cavity is blue detuned, the lower polariton branch becomes more exciton-like and hence shows a reduced and shorter-lived superdiffusive expansion.

### Renormalization of polariton velocity

**Fig. 4.**
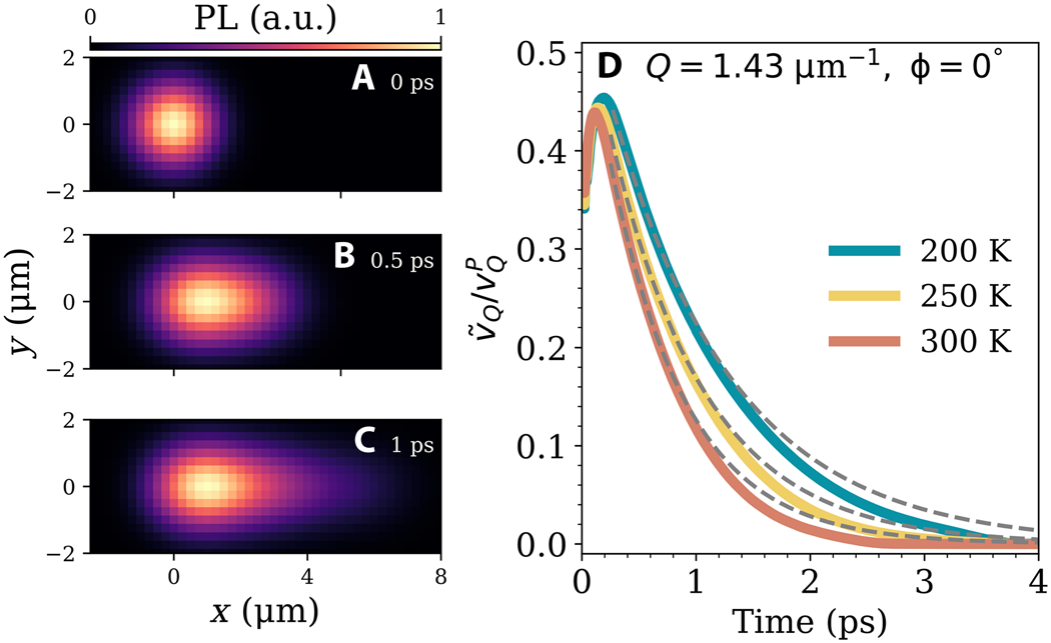
Angle-resolved polariton transport. Ballistic-like to superdiffusive transport regime. (**A** to **C**) PL maps of the momentum- and angle-resolved polariton density shown at the fixed momentum *Q* = 1.43 μm^−1^ (corresponding to the maximum group velocity $v_{Q}^{P}$, see Fig. 2, B and F) and in-plane angle ϕ = 0° for three fixed times. (**D**) Ratio of the effective velocity, ${\tilde{v}}_{Q}=d{\langle x\rangle }_{Q}\;/\;\mathrm{dt}$, and the group velocity, $v_{Q}^{P}$, at three different temperatures. The gray dashed lines show a simple decay model based on the probability that a polariton has scattered with a phonon or radiatively decayed into an external photon.

Now, we further explore the ballistic-like and the superdiffusive transport regime by studying the momentum- and angle-resolved polariton density, i.e., polariton propagation in a particular in-plane direction. This is an experimentally relevant quantity that is linked to the PL measured at a distance away from the excitation point, which selectively probes polaritons that have propagated in a particular direction (*33*, *35*, *41*). Figure 4 (A to C) shows a typical PL density map during the first picosecond of expansion, for an in-plane angle = 0° and momentum *Q* = 1.43 μm^−1^, corresponding to the maximum group velocity $v_{Q}^{P}$ (blue line in Fig. 2F). It highlights a rapid propagation of angle-resolved polaritons over micrometers, even at room temperature and in the presence of strong polariton-phonon scattering.

To quantify directional polariton transport, we introduce the first moment of the exciton polariton cloud, ${\langle x\rangle }_{Q}=\int d^{2}R\;N_{Q}\left(R,t\right)x/\int d^{2}R\;N_{Q}\left(R,t\right)$, and take the time derivative to give an effective velocity, ${\tilde{v}}_{Q}=d{\langle x\rangle }_{Q}/\mathrm{dt}$. In the limit of no scattering with phonons, this will simply be the group velocity extracted from the polariton dispersion (see fig. S2). In Fig. 4D, the ratio ${\tilde{v}}_{Q}/v_{Q}^{P}$ is shown for three different temperatures at *Q* = 1.43 μm^−1^ (different momenta can be found in fig. S4). For all temperatures, the effective velocity is strongly renormalized because of scattering with phonons, even in the subpicosecond ballistic-like regime. On an ultrashort timescale of 100 fs, we find a small rise of the effective velocity, which is attributed to the nonresonant excitation. We find that the effective group velocity of polaritons is quite sensitive to the initial conditions of the system (see fig. S7). Unexpectedly, we observe that the maximum effective velocity is quite temperature-independent reaching a value of about $0.45\times v_{Q}^{P}$ for all three temperatures considered. This is a consequence of the weak temperature dependence of the in-scattering from the initial hot-exciton distribution.

The decrease in ${\tilde{v}}_{Q}$ over time is much less efficient at lower temperatures. For example, at 3 ps the effective velocity of $0.019\times v_{Q}^{P}$ at 200 K is about 90 times larger than the respective room temperature value. This can be understood using a simple model that describes the probability over time of a polariton undergoing a scattering event or radiatively decaying: $x_{Q}\left(t\right)=\int_{0}^{t}dt^{\prime }\;\text{exp}\left[-\left(\Gamma_{Q}+\gamma_{Q}\right)t^{\prime }\right]v_{Q}^{P}$, suggesting the identification of an effective velocity ${\tilde{v}}_{Q}\left(t\right)\propto \text{exp}\left[-\left(\Gamma_{Q}+\gamma_{Q}\right)t\right]v_{Q}^{P}$. We find that this provides a reasonable fit to the results (dashed gray lines in Fig. 3D), especially considering that this model does not account for the generation of new polaritons from the (nearly) static exciton reservoir. This explains why the model underestimates the drop in velocity with time. It does not take into account the generation of polaritons at later times within the original excitation area, which anchors 〈x〉 to this region and hence reduces the effective velocity. This can be seen in Fig. 4C, where the central region emits the most intense PL.

### Polariton-mediated transport of dark excitons

**Fig. 5.**
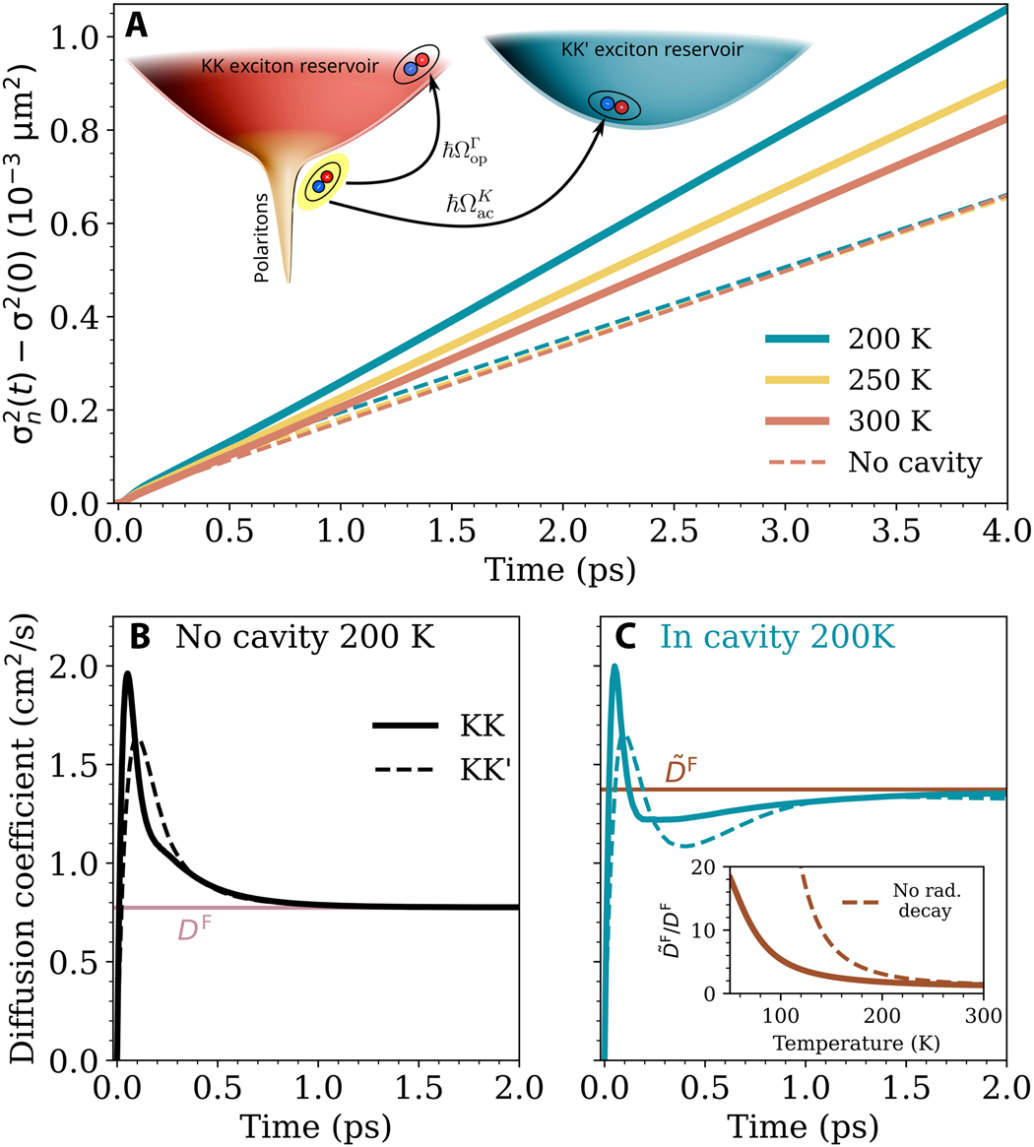
Cavity-enhanced exciton reservoir diffusion. (**A**) Broadening of the total momentum-integrated KK-exciton-polariton density at three different temperatures (including all momenta inside and outside the lightcone). Solid (dashed) lines show the case with (without) a cavity. This reveals an enhanced polariton-mediated exciton propagation in a cavity, as illustrated by the schematic in the inset. (**B** and **C**) The corresponding valley-resolved diffusion coefficients for the system with and without a cavity, respectively. The horizontal lines denote the respective steady-state diffusion coefficient, *D^F^*, estimated with Fick’s law. The inset illustrates the temperature-dependent cavity enhancement effect, where the dashed line shows the result in the absence of polariton radiative decay.

Thus far, we have focused on the propagation of polaritons with momenta within the lightcone. We now consider the total momentum-integrated Wigner function over all states, both inside and outside the lightcone, and focus on the third transport regime: exciton-limited steady-state diffusion. Figure 5A shows the broadening of the entire KK exciton polariton cloud at three different temperatures (equivalent detuning study can be found in fig. S6). The corresponding dashed lines illustrate the broadening in a bare exciton system without a cavity. Note that the dashed lines coincide closely, highlighting the relative temperature insensitivity of the bare excitons in contrast to the cavity system (see fig. S8). For both systems and all three temperatures, from about 0.5 ps onward we find that the broadening has an approximately linear time dependence, indicating a rapid thermalization. Here, the transport is dominated by conventional Fickian diffusion (*64*). Given the tiny DOS of polaritons within the lightcone, it might be expected that the total broadening would be unchanged in the presence of a cavity. Unexpectedly, Fig. 5A reveals that the expansion significantly speeds up in the presence of a cavity, indicating a cavity enhancement effect in the steady-state (*60*). This effect is the strongest at the lowest temperature of 200 K. For example, at 4 ps the exciton cloud has expanded 160% more at 200 K compared to the cavity-free case, while this is 125% at 300 K. The mechanism behind this polariton-mediated transport enhancement is illustrated in the inset of Fig. 5A. At elevated temperatures, rapid polaritons in the lightcone can scatter into both the KK and KK′ exciton reservoir via phonon absorption. This can happen away from the initial excitation region after a polariton has traveled some distance, leading to an effective transport of the exciton reservoir. While phonon absorption becomes weaker at lower temperatures, the combined effects of increased polariton occupation relative to the exciton reservoir and faster polariton expansion (see Fig. 3A) lead to a stronger cavity-enhanced expansion.

Turning now to the corresponding effective diffusion coefficients, Fig. 5 (B and C) shows the case without and with a cavity at 200 K, respectively. Here, we plot both the KK (solid) and KK′ (dashed) valley-resolved diffusion coefficients, which at thermal equilibrium will limit to the same value due to efficient phonon-mediated intervalley scattering (see fig. S5). Below 0.5 ps, we find in all cases a sharp transient peak in the diffusion due to the initial hot-exciton population (*64*) that is only weakly affected by the presence of a cavity. In contrast, at the steady state, the presence of a cavity leads to an increased diffusion for both valleys (for KK this corresponds to the different slopes of the solid and dashed teal lines in Fig. 5A). For the bare exciton case, the steady state value can be predicted using Fick’s law (*64*, *65*), $D_{n}^{F}=\left(\hbar /2\right)\sum_{Q}f_{\mathrm{nQ}}v_{\mathrm{nQ}}^{2}\Gamma_{\mathrm{nQ}}^{-1}/N_{n}$, which holds for a quasi-thermalized local distribution where $f_{\mathrm{nQ}}$ is the Boltzmann distribution for the valley *n*, and $N_{n}=\sum_{Q}f_{\mathrm{nQ}}$. In a two-valley system, the total steady-state diffusion coefficient is given by the population-weighted valley-resolved coefficients, $D^{F}=\left(D_{\text{KK}}^{F}N_{\text{KK}}+D_{{\text{KK}}^{\prime }}^{F}N_{{\text{KK}}^{\prime }}\right)/\left(N_{\text{KK}}+N_{{\text{KK}}^{\prime }}\right)$. This is shown in Fig. 5B with the horizontal pink line, where *D*^F^ = 0.77 cm^2^/s, which correctly gives the steady-state limit of the full Boltzmann transport dynamics. Fick’s law can also be applied to polaritons (*60*), but it is important to correct for the bottleneck effect (*53*). In the limit of not too strong radiative decay, we can take a corrected Fick’s law, ${\tilde{D}}_{\text{KK}}^{F}$, with $\Gamma_{\mathrm{nQ}}\to \Gamma_{\mathrm{nQ}}+\gamma_{\mathrm{nQ}}$, and ${\tilde{f}}_{\mathrm{nQ}}=\Gamma_{\mathrm{nQ}}/\left(\gamma_{\mathrm{nQ}}+\Gamma_{\mathrm{nQ}}\right)f_{\mathrm{nQ}}$ as the modified occupation factor (see the Supplementary Materials for further details). Because of the contribution of the high-velocity polaritons, we find that ${\tilde{D}}^{F}$ has a much higher value of 1.37 cm^2^/s compared to *D*^F^, shown by the brown horizontal line in Fig. 5C. This compares to 2.32 cm^2^/s, if the polariton occupation is incorrectly assumed to follow a Boltzmann distribution. Figure 5C reveals that the full dynamics of both the KK and KK’ exciton reservoirs correctly limit toward the polariton-modified Fick’s law as the system thermalizes, exhibiting a cavity-induced enhancement of ${\tilde{D}}^{F}/D^{F}=1.77$. Given the excellent agreement between the polaritonic Fick’s law and the steady-state limit of the numerical results, we can explore the cavity enhancement over a large temperature range at greatly reduced computational cost. The inset of Fig. 5C reveals that at lower temperatures, the cavity enhancement of the diffusion coefficient grows rapidly, reaching 18.3 at 50 K. This stems from a higher polariton occupation relative to the reservoir and an increased mean free path of the excitons/polaritons. In the absence of radiative decay (dashed line), there is no polariton bottleneck and an even larger diffusion enhancement is possible, reaching ~10^4^ at 50 K (*60*). While the impact of the exciton reservoir on spatiotemporal polariton dynamics is established in the literature (*53*, *70*), we show here that also a polariton population measurably influences the exciton reservoir, despite its small DOS. This prediction is directly testable via phonon sidebands (*71*) and pump-probe techniques (*72*).

## DISCUSSION

In this work, we provide microscopic insights into ultrafast transport phenomena of exciton-polaritons in 2D semiconductors, crucially taking into account the full exciton energy landscape. We find three distinct transport regimes, ranging from the initial subpicosecond ballistic-like regime with phonon-induced renormalization of the effective polariton velocity, to a few-picosecond superdiffusive regime with a massive polariton-enhanced diffusion, and a slower steady-state conventional exciton diffusion. For the latter, we predict a polariton-mediated enhancement of exciton reservoir diffusion via phonon-driven re-population of higher energy dark excitons from highly mobile polaritons. The results highlight the importance of polariton-phonon scattering at ambient temperatures, as well as of the crucial role of the entire exciton reservoir as scattering partners for polaritons. The developed approach can be applied to a broader class of excitonic 2D materials, such as metal-halide perovskites (*32*), as well as to spatially structured cavities that host, for example, trapped (*33*) and lattice (*73*) polaritons. In addition, our approach is applicable to studying waveguide polariton transport (*36*, *40*, *74*). These high-velocity, nonradiative modes are naturally attractive for on-chip polaritonic circuitry. Moving beyond the low-excitation regime, optical techniques can be used to shape the effective potential experienced by polaritons, for example providing directional transport (*55*). Moreover, by clarifying the fundamental role of the exciton reservoir and phonon scattering, our work could also be adapted to explore generation and propagation of polariton condensates (*27*, *75*).

## METHODS

Using a material-specific Wannier-Hopfield method (*59*), we calculate the dynamics of the space- and momentum-resolved polariton Wigner function (*63*), $N_{nQ}\left(R,t\right)$, using the Heisenberg equation of motion (*76*). Here, *n* denotes both the polariton branch and the exciton valley, while Q is the center-of-mass momentum and R the spatial coordinate. We restrict our focus to the low-excitation regime, where the dominant source of scattering is exciton/polariton-phonon scattering. In analogy to the bare exciton case (*21*, *64*, *65*), the resulting spatiotemporal dynamics is described by a Boltzmann transport equation (see the Supplementary Materials for further details)

$$\begin{array}{c} \begin{array}{l} \partial_{t}N_{nQ}\left(R,t\right)=\left(-v_{nQ}\cdot \nabla -2\Gamma_{nQ}-2\gamma_{nQ}\right)N_{nQ}\left(R,t\right)\\ +\sum_{mQ^{\prime }}\Gamma_{mQ^{\prime },nQ}N_{mQ^{\prime }}\left(R,t\right)\; \end{array} \end{array}$$ (1)

where $v_{nQ}=\partial_{Q}E_{nQ}/\hbar$ is the group velocity, $\gamma_{nQ}$ the radiative decay rate, $\Gamma_{nQ}$ the exciton/polariton-phonon dephasing rate, and $\Gamma_{mQ^{\prime },nQ}$ the in-scattering rate from the state $\langle m,Q^{\prime }\rangle$ into 〈n,Q〉 via emission or absorption of phonons. The first term in Eq. 1 describes ballistic motion, where each state moves unimpeded through space with the velocity $v_{nQ}$. Exciton/polariton-phonon scattering is treated on the level of the second-order Born-Markov approximation (*51*–*53*, *60*). For polaritons, this is dominated by scattering into exciton states outside the lightcone.

The exciton-microcavity coupling is modeled by taking into account penetration of the cavity field into the DBR mirrors (see the Supplementary Materials for details). The latter are taken to be eight periods of SiO_2_ and NbO_2_, giving a reflectance of 98.7% at the center of the stopband. On the basis of calculations of the spatially homogeneous dynamics (*53*), the upper polariton is found to have a negligible contribution and is hence ignored. To describe nonresonant excitation of the system, all dynamical calculations are initialized with a Gaussian distribution in the KK exciton reservoir, centered at 50 meV and with a width of 1 meV. To mimic the excitation of the system by a laser with a typical finite beam waist, the spatial distribution of the polariton cloud is initialized as a Gaussian with a FWHM of 2 μm. The results are found not to be sensitive to the choice of this width. Because of the system’s translational invariance, the exciton-polariton cloud remains rotationally invariant at all times.

## Supplementary Materials

### This PDF file includes:

Sections S1 to S10 Figs. S1 to S8 References
