# Supplementary material for: Polariton transport in 2D semiconductors: Phonon-mediated transitions between ballistic, superdiffusive, and exciton-limited regimes
Source: Sci Adv. 2025 Nov 14;11(46):eaea3495. doi: 10.1126/sciadv.aea3495 (PMC13142775; doi:10.1126/sciadv.aea3495)
Supplement: Supplementary file 1 — Sections S1 to S10 Figs. S1 to S8 References [file sciadv.aea3495_sm.pdf]

Science Advances
AAAS

Supplementary Materials for

Polariton transport in 2D semiconductors: Phonon-mediated transitions between ballistic, superdiffusive, and exciton-limited regimes

Jamie M. Fitzgerald *et al.*

Corresponding author: Jamie M. Fitzgerald, jamie.fitzgerald@physik.uni-marburg.de

Sci. Adv. **11**, eaea3495 (2025)
DOI: 10.1126/sciadv.aea3495

This PDF file includes:

Sections S1 to S10
Figs. S1 to S8
References

S.I Theoretical Methods

S.I.1 Modelling excitons with the Wannier equation

Exciton energies, wavefunctions, and oscillator strengths are calculated using the Wannier equation (*56, 77*), with density functional theory (DFT) input used to characterise the two-band parabolic approximation for the electronic bandstructure (*78*). The Wannier equation is solved for the bright KK, the momentum-dark KK', and KΛ 1s excitons. The latter are found to be approximately 140 meV higher in energy than the KK excitons, and hence have a negligible contribution to the dynamics explored in this work. The spectral position of KK excitons in hBN-encapsulated $MoSe_2$ monolayers is fixed to 1.65 eV to match PL measurements (*58*). The screened Coulomb potential is modelled as a generalised Keldysh potential (*79*) using DFT-derived dielectric constants for the TMD monolayer ($\epsilon_\perp = 7.2$ and $\epsilon_{||} = 16.8$) (*80*), and $\epsilon_{\text{sub}} = 4.5$ for the surrounding hBN layers.

To estimate the optical matrix element, which dictates the strength of the exciton-photon coupling and the Rabi splitting in a cavity, a two-band $\mathbf{k}\cdot\mathbf{p}$ expansion is used (*81*), $M_0 = m_0\sqrt{E_g/(4m_r)}$. Here, $m_r = m_e m_h/(m_e + m_h)$ is the reduced electron-hole mass, m_0 is the free electron mass, and $m_{e/h}$ the effective electron/hole mass. The exciton radiative lifetime is given by (*76*)

$$\gamma^X = \frac{e^2 M_0^2 |\psi^X(\mathbf{r}=0)|^2}{2m_0^2 \epsilon_0 c E_0^X}, \tag{S1}$$

where E_Q^X and ψ^X are the 1s KK exciton energy and wavefunction, respectively.

S.I.2 Exciton-photon coupling in a cavity

We focus on the lowest energy optical mode of a symmetric $\lambda/2$ Fabry-Pérot microcavity in vacuum, where a single-mode approximation is valid due to the large free-spectral range. For small microcavities constructed from distributed Bragg reflectors (DBRs), it is essential to account for the phase penetration of the cavity field into the mirrors to accurately calculate the cavity mode dispersion and the exciton-cavity coupling. For frequencies close to the stop-band centre frequency, ω_s, the reflection coefficient has a linear dependence on phase, leading to a simple expression for the penetration depth (*82*)

$$L_{\text{DBR}} = \frac{\lambda_s/2}{n_H - n_L}, \tag{S2}$$

where $\lambda_s = 2\pi c/\omega_s$, and $n_{L/H}$ are refractive indices of the alternating low/high-index layers of the DBR. This expression is valid for a DBR where the first interface is the high-index material. For the alternative situation, where the first interface is the low-index material, the penetration depth is given by $(n_L n_H \lambda_s/2)/(n_H - n_L)$ (*82*). Assuming frequencies close to the stop-band, the modified resonance condition of the cavity can be written as (*83*)

$$\tilde{E}^c(\theta) = \frac{L_c E^c(\theta) + L_{\mathrm{DBR}}\hbar\omega_s(\theta)}{L_c + L_{\mathrm{DBR}}}, \tag{S3}$$

where θ is the angle of incidence, L_c is the physical cavity length, and $E^c(\theta) = \hbar\pi c/(L_c\cos(\theta))$ is the Fabry-Pérot mode energy in the absence of any phase delay. The angle θ also corresponds to the photon propagation angle within the cavity, as we assume a vacuum for both the external and internal media. A simple expression for the angle-dependent stop-band frequency can be derived by considering the phase change across one period of the DBR

$$\omega_s(\theta) = \frac{2\omega_s(0)}{\cos(\theta_L) + \cos(\theta_H)}, \tag{S4}$$

where $\theta_{L/H}$ are the angles of propagation in the low/high-index layers, which can be determined from Snell's law. The angle can linked to the momentum of a particle via the relation $Q = \sin(\theta)E(\theta)/(\hbar c)$, where E is the particle's dispersion. Figure S1 shows a comparison of the model (dashed purple curve) against the commonly used perfect-mirror model ($L_{\mathrm{DBR}} = 0$, dashed brown curve), and the exact cavity mode dispersion calculated with the T-matrix method (*32*) (dashed blue curve). Excellent agreement is found at small angles/momenta.

The cavity decay rate is estimated as (*59*)

$$\hbar\kappa = \frac{\hbar c(1 - R_s)}{2(L_c + L_{\mathrm{DBR}})} \tag{S5}$$

with (*83*) $R_s \approx 1 - 4\left(\frac{n_L}{n_H}\right)^{2N}$, where N is the number of periods in the DBR. The exciton-cavity coupling depends on the polarization for non-zero angles. In this work we focus exclusively on TM polarization, yielding (*59, 83*)

$$g \approx \sqrt{\frac{2c\hbar\gamma^X}{L_c + L_{\mathrm{DBR}}}}, \tag{S6}$$

which is angle/momentum independent. For the 8-period NbO_2/SiO_2 DBR considered in this work, we find a Rabi splitting of $2g = 39$ meV, in good agreement with experimental values from similar

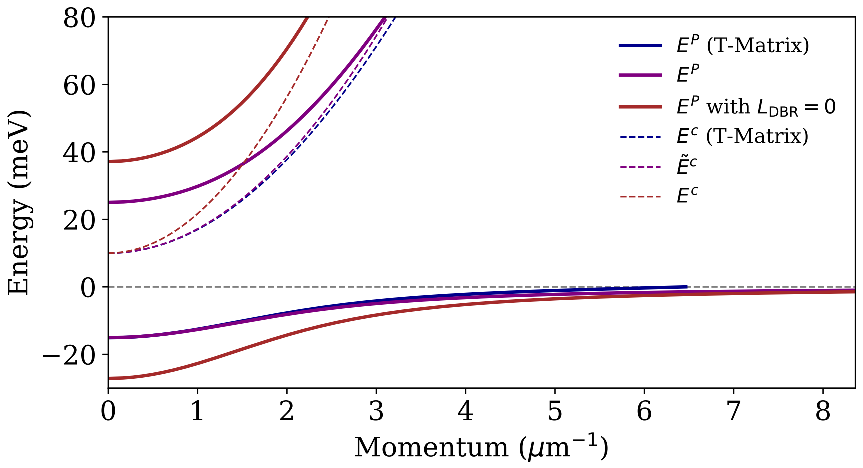

Figure S1: **Comparison of polariton and cavity dispersion models.** Energy (relative to the 1s KK exciton) of the lower and upper polaritons (solid lines), and cavity mode energies (dashed lines) for a 10 meV blue detuned $\lambda/2$ cavity with a MoSe2 monolayer in the centre. The end mirrors are two identical 8-period NbO_2/SiO_2 DBRs with a stop-band centre reflectance of 98.7%. The dashed and solid brown lines show the cavity, E^c, and polariton energy, respectively, within the perfect-mirror model (no phase penetration into the DBR, $L_{\text{DBR}} = 0$). Likewise, $\tilde{E}^c$ (dashed purple curve) and E^P (solid purple curve) give the cavity and polariton energy, respectively, including corrections for the DBR phase penetration. Excellent agreement between this model and the T-matrix method (blue curves) is found. Note that the T-matrix fit for the lower polariton energy is terminated at the start of the leaky-mode region (*54*), where the single optical mode assumption breaks down.

microcavity systems (*84*). This compares to a value of 63.5 meV using the perfect-mirror model. As seen for the lower polariton in Fig. S1, the corrected model (purple curve) gives a much better agreement with the extracted T-matrix dispersion (blue curve) when compared to the perfect-mirror model (brown curve).

An advantage of the model described here is that all quantities can be calculated analytically from the DBR parameters, eliminating the need for full-wave simulation of Maxwell's equations, such as the T-matrix method (*32*). Furthermore, it is simple to utilize the Hopfield method (i.e., a coupled oscillator model) to describe the strong coupling between the bright KK exciton and the cavity photon. The polariton energies and eigenfunctions (Hopfield coefficients) can be expressed

analytically for a one-exciton/one-photon system. For the lower polariton branch we find (*81*)

$$E^P_{\mathbf{Q}} = \frac{E^c_{\mathbf{Q}} + E^X_{\mathbf{Q}}}{2} - \frac{1}{2}\sqrt{(E^c_{\mathbf{Q}} - E^X_{\mathbf{Q}})^2 + 4g^2} \tag{S7}$$

$$U_{\mathbf{Q}} = \sqrt{\frac{E^P_{\mathbf{Q}} - E^X_{\mathbf{Q}}}{2E^P_{\mathbf{Q}} - E^X_{\mathbf{Q}} - E^c_{\mathbf{Q}}}} \tag{S8}$$

$$V_{\mathbf{Q}} = \sqrt{\frac{E^P_{\mathbf{Q}} - E^c_{\mathbf{Q}}}{2E^P_{\mathbf{Q}} - E^X_{\mathbf{Q}} - E^c_{\mathbf{Q}}}}, \tag{S9}$$

where $U_{\mathbf{Q}}/V_{\mathbf{Q}}$, give the photonic/excitonic character of the exciton polariton. The resulting polariton radiative decay rate is given by the bare cavity decay rate scaled by the photonic Hopfield coefficient (see the green shaded curve in the inset of Fig. 2(c) in the main text)

$$\hbar\gamma^P_{\mathbf{Q}} = |U_{\mathbf{Q}}|^2\hbar\kappa. \tag{S10}$$

In principle, the coupling and hence Hopfield coefficients can be extracted from a T-matrix simulation, giving exact results at the level of Maxwell's equations (*32*). In practice, the issue with this approach is that other photonic modes (in particular, leaky modes of the DBR) can interact with the exciton, disrupting the fitting procedure at large angles. This could be overcome by including additional photonic modes in the Hopfield method, but the computational cost would rise dramatically. Because the model neglects leaky modes, it fails to account for large-angle leakage of the Bragg mirrors (*54*), thereby underestimating of the polariton radiative decay at high momenta within the lightcone.

S.I.3 Exciton- and polariton-phonon coupling

Exciton- and polariton-phonon scattering is taken into account by considering acoustic (longitudinal and transverse) and optical (longitudinal, transverse, and the out-of-plane A_1) phonons at the high-symmetry points of the phonon dispersion (*61*). Phonons at these momenta are relevant for phonon-driven intra- and intervalley exciton scattering (*53*). For simplicity and to ease the computational burden, the set of phonons at each high-symmetry point is treated as one averaged acoustic and one averaged optical phonon (*85*). The relevant phonon modes for this work are the Γ phonons, which drive intravalley scattering, and *K* phonons, which can scatter conduction electrons between the K and K′ valley of the electronic bandstructure to create KK′ excitons. In particular, coherent

phonon studies have shown that longitudinal acoustic K phonons are responsible for the strong valley depolarization observed in $MoSe_2$ monolayers (*62*). The long-range Γ acoustic phonons are approximated with a linear dispersion (Debye model), while all other phonons are treated with a constant energy approximation (Einstein model).

Electron-phonon scattering matrix elements, $D_{\mathbf{q}}^{c/v}$, are determined within the deformation potential approximation, with relevant parameters taken from DFT studies (*61*). Solving the Wannier equation and converting to the exciton basis then gives the exciton-phonon matrix elements (*57, 77, 79*). The relative phases of the electron-phonon matrix elements are not specified in Ref. 61, but they are important for determining the strength of the intravalley exciton-phonon coupling, which depends on the difference of the electron and hole coupling to phonons. They must be inferred from the dominant physical scattering mechanism for each phonon species (*86*). Considering acoustic modes, we assume that the coupling strength predominantly arises from a non-polar lattice deformation, meaning that the conduction and valence band shift in opposite directions under strain ($|D_{\mathbf{q}}^{c} + D_{\mathbf{q}}^{v}|$). In contrast, optical modes are assumed to predominantly couple to electrons via the Fröhlich interaction, where the two bands shift in the same direction energetically ($|D_{\mathbf{q}}^{c} - D_{\mathbf{q}}^{v}|$) (*86, 87*). While the averaging over phonon modes employed in this work leads to a simpler picture of exciton-phonon interactions, it risks inaccurately estimating the exciton-phonon coupling if there is a mix of different mechanisms. In particular, the intravalley scattering of excitons via Γ optical phonons is sensitive to the choice of phase.

After performing a Hopfield transformation, the polariton-phonon scattering strength is given by the exciton-phonon matrix element, $D_{\alpha,\mu\nu,q}$, scaled by the excitonic component of both the initial and final polariton state: (*51–53, 60, 85*) $\tilde{D}_{\alpha,n\mathbf{Q},m\mathbf{Q}'} = \sum_{\mu\nu} V^{*}_{\mu n,\mathbf{Q}} D_{\alpha,\mu\nu,|\mathbf{Q}'-\mathbf{Q}|} V_{\nu m,\mathbf{Q}'}$. Here, $q = |\mathbf{Q} - \mathbf{Q}'|$ is the momentum imparted by the phonon, and α is the phonon species index. These matrix elements describe both the strength of intravalley scattering within the polariton branches (including the dark reservoir excitons outside the lightcone), and scattering between dark intervalley excitons and polaritons.

S.II Polariton Boltzmann transport equation

Now, we investigate polariton dynamics in a spatially homogeneous system that has an initial inhomogeneous spatial distribution, such as a polariton gas generated by a laser with a finite beam waist. We begin by introducing a polaritonic density matrix, $\rho_{n\mathbf{Q},m\mathbf{Q}'} = \langle \hat{P}_{n\mathbf{Q}} \hat{P}_{m\mathbf{Q}'} \rangle$, which contains information about the spatial distribution of polaritons in the off-diagonal and their occupation in the diagonals. In direct analogy with the bare exciton case (*64, 65*), we integrate out relative coordinates of the polariton/exciton and define a polaritonic Wigner function for the nth branch/valley

$$N_{n\mathbf{Q}}(\mathbf{R}) = \sum_{\mathbf{q}} \langle \hat{P}^{\dagger}_{n\mathbf{Q}+\mathbf{q}} \hat{P}_{n\mathbf{Q}} \rangle \exp[i\mathbf{q} \cdot \mathbf{R}], \tag{S11}$$

which gives the spatially dependent quasi-probability distribution for a polariton.

To derive the Boltzmann transport equation for the polariton Wigner function, we start by considering the semi-classical dynamics of a particle at a spatial region $\{\mathbf{R}, \mathbf{Q}\}$, and in a band $E_{n\mathbf{Q}}$ over an infinitesimal time dt (*88*). In the absence of external forces, the coordinates evolve as $\{\mathbf{R}, \mathbf{Q}\} \to \{\mathbf{R} + \mathbf{v}dt, \mathbf{Q}\}$, where $\mathbf{v}_{n\mathbf{Q}} = \partial_{\mathbf{Q}} E_{n\mathbf{Q}}/\hbar$ is the group velocity. Collision processes (in this case via lattice vibrations) and radiative decay result in a net rate of change in the number of particles in the phase space region $d\mathbf{R}d\mathbf{Q}$. Using the Liouville theorem, a condition of detailed balance can be expressed for each polariton/exciton branch/valley

$$\begin{aligned} N_{n\mathbf{Q}}(\mathbf{R} + \mathbf{v}dt, t + dt) &= N_{n\mathbf{Q}}(\mathbf{R}, t) + \partial_t N_{n\mathbf{Q}}(\mathbf{R})|_{\text{scat}} dt + \partial_t N_{n\mathbf{Q}}(\mathbf{R})|_{\text{rad}} dt \\ \therefore\ \frac{\partial}{\partial t} N_{n\mathbf{Q}}(\mathbf{R}, t) + \mathbf{v}_{n\mathbf{Q}} \cdot \nabla N_{n\mathbf{Q}}(\mathbf{R}, t) &= \partial_t N_{n\mathbf{Q}}(\mathbf{R}, t)|_{\text{scat}} + \partial_t N_{n\mathbf{Q}}(\mathbf{R}, t)|_{\text{rad}}, \end{aligned} \tag{S12}$$

where in the second line we expand to first order (*88*). The second term on the left describes ballistic propagation (free evolution) of polaritons, where each state moves along the direction of $\mathbf{Q}$ at the group velocity $\mathbf{v}_{nQ}$. Using an equation of motion approach (*76, 89*) and assuming a low polariton/exciton density, the scattering terms, $\partial_t N_{n\mathbf{Q}}(\mathbf{R}, t)|_{\text{scat}}$ and $\partial_t N_{n\mathbf{Q}}(\mathbf{R}, t)|_{\text{rad}}$, can be derived by considering the coupling of polaritons to a thermalized phonon bath and external photon ports (quasi-mode approximation), respectively. This procedure is described in detail in Ref. 53, so we quote only the final result here (equation (1) of the main text)

$$\frac{\partial}{\partial t} N_{n\mathbf{Q}}(\mathbf{R}, t) = (-\mathbf{v}_{n\mathbf{Q}} \cdot \nabla - 2\Gamma_{n\mathbf{Q}} - 2\gamma_{n\mathbf{Q}}) N_{n\mathbf{Q}}(\mathbf{R}, t) + \sum_{m\mathbf{Q}'} \Gamma_{n\mathbf{Q},m\mathbf{Q}'} N_{m\mathbf{Q}'}(\mathbf{R}, t). \tag{S13}$$

For a high-quality symmetric cavity, the polariton decay rate is given by Eq. S10, i.e., the total cavity decay rate scaled by the photonic Hopfield coefficient (*53*). The exciton/polariton-phonon scattering rate is calculated on the level of the Born-Markov approximation, describing the probability for a phonon to scatter an exciton/polariton from state $|n\mathbf{Q}\rangle$ to $|m\mathbf{Q}'\rangle$. It is given by:

$$\Gamma_{n\mathbf{Q},m\mathbf{Q}'} = \frac{2\pi}{\hbar} \sum_{\alpha,\pm} \left|\tilde{D}_{\alpha,n\mathbf{Q},m\mathbf{Q}'}\right|^2 \left(\frac{1}{2} \pm \frac{1}{2} + n^{\text{phn}}_{\alpha,|\mathbf{Q}-\mathbf{Q}'|}\right) \delta\left(E_{m\mathbf{Q}'} - E_{n\mathbf{Q}} \pm E^{\text{phn}}_{\alpha,|\mathbf{Q}-\mathbf{Q}'|}\right). \tag{S14}$$

Here, the phonon occupation factor, $n^{\text{phn}}_{\alpha\mathbf{q}}$, is given by a Bose Einstein distribution (phonon bath approximation), $\pm$ denotes phonon emission/absorption, $E^{\text{phn}}_{\alpha,q}$ is the phonon energy, and the delta function enforces strict energy and momentum conservation. The corresponding out-scattering term is given by summing over all possible energy- and momentum-conserving channels: $\Gamma_{n\mathbf{Q}} = \sum_{m\mathbf{Q}'} \Gamma_{n\mathbf{Q},m\mathbf{Q}'}/2$. This leads to openings of scattering channels at certain momenta of the polariton dispersion (see Fig. 2(c) in the main text). Neglected higher-order dephasing processes are expected to broaden the opening of scattering channels (*90*). It is the exciton/polariton-phonon scattering terms of Eq. S13 that drive diffusion in the system. They redistribute the polariton occupation primarily toward lower energy states (energy-relaxation) and different orientations in reciprocal space (momentum-relaxation) (*65*).

Solving Eq. S13 poses a challenging numerical problem due to the large range of momentum and spatial scales that must be resolved, spanning from photonic phenomena on the scale of micrometers to excitonic phenomena at the nanometre scale. For the discretisation in momentum space, we use the same method as introduced in Ref. 53. We find that using a logarithmic momentum grid works well at accurately describing the dynamics of both polaritons within the lightcone and the exciton reservoir outside. Particle conservation is not enforced and can be used as a check of the numerical calculation. In principle, polariton population is lost due to radiative recombination within the lightcone, but these states represent a tiny fraction of the total KK exciton reservoir, meaning that the total polariton number is approximately conserved over the timescales we explore. We solve the spatiotemporal dynamics on a two-dimensional Cartesian grid using a second-order centred finite-difference approximation. To mimic non-resonant excitation, all dynamics (unless otherwise stated) are initialized with a Gaussian distribution of width 1 meV and centred at 50 meV in the KK reservoir. A study of the impact of the initialization energy on the results is presented in Section S.VIII.

S.III Pure ballistic polariton transport

In the absence of phonon scattering, $\Gamma_{nQ,mQ'} = 0$, the Boltzmann equation reduces to a purely ballistic equation that can be solved independently for each momentum (i.e., a collection of two-dimensional advection equations). In Fig. S2(a), the ballistic motion of a single momentum ($1.43\ \mu\text{m}^{-1}$, corresponding to maximum v_Q^P) and angular component of the polariton Wigner function is shown at three different times. It is a shape-preserving evolution; it remains a Gaussian at all times, with only a change in amplitude due to radiative decay and a shift in the offset. Therefore, it is exactly described by a time-dependent Gaussian (dashed black line):

$$N_Q(x, y = 0, t) \propto \exp\left(-\frac{(x - v_Q^P t)^2}{2\sigma_0^2}\right) \exp(-\gamma_Q t). \tag{S15}$$

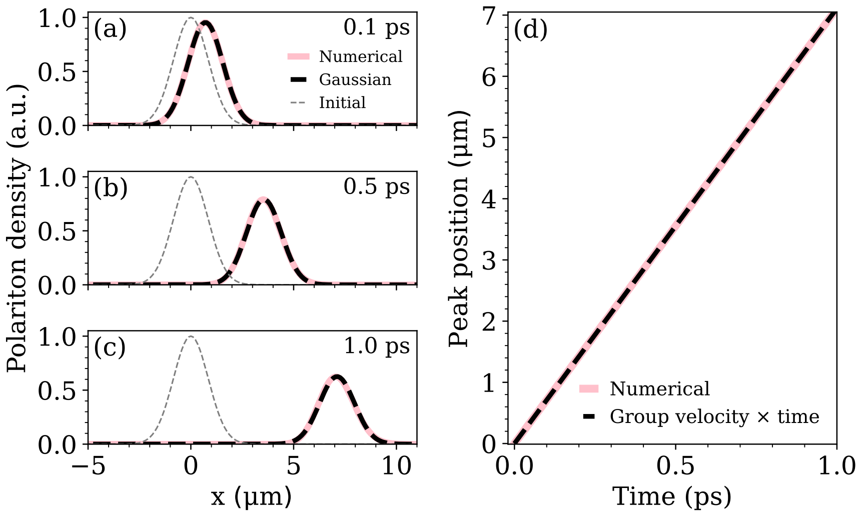

Figure S2: **Ballistic transport of exciton polaritons in the absence of phonon scattering.** (a)-(c) Slices of the angle- and momentum-resolved polariton density at three successive times. Evaluated for a momentum of $Q = 1.43\ \mu\text{m}^{-1}$ (corresponding to the maximum of the group velocity v_Q^P), assuming no phonon scattering and a +10 meV detuned cavity. The thin dashed grey line shows the initial Gaussian distribution, while the pink curve is the numerical result from solving Eq. S13 with $\Gamma_{nQ,mQ} = 0$. The dashed black line shows the analytical result (Eq. S15). The decay in the Gaussian's height with time is due to radiative decay. (b) The peak position of the Gaussian extracted from the first moment, $\langle x \rangle_Q$ (pink line), and calculated analytically with $v_Q^P t$ (dashed black line).

In Fig. S2(b), the peak position of the Gaussian is calculated with the first moment (pink line), $\langle x\rangle_Q = \int dx\, xN_Q(x, y = 0, t)/\int dxN_Q(x, y = 0, t)$, which can be compared to the analytical result (dashed black line) for the time-dependent offset, $v_Q^P t$, where perfect agreement is found. This illustrates that, without phonon scattering, polaritons move ballistically at the group velocity.

S.IV Momentum-resolved diffusion coefficients

Figure S3 shows the momentum-resolved effective diffusion coefficients (i.e., the time derivative of the broadening, $D(t) = \partial_t\sigma^2(t)/4$) at 300 K and $\Delta = +10$ meV for the five representative momenta considered in Fig. 2(f) of the main text (dashed coloured lines in Figs. 2(b) and (c)). For the first three momenta considered (0.5, 1.0 and 1.43 μm^{-1}), the peak diffusion increases with the group velocity (see Fig. 2(b)), but the time at which the maximum is reached is roughly the same for all three momenta. This is a consequence of the approximately constant phonon scattering rate over this range of momenta (see the inset of Fig. 2(c)). The effective diffusion coefficients for the next two momenta (2 and 5 μm^{-1}) exhibit a different behaviour. The peak diffusion value is shifted to earlier times and reduced in magnitude due to the opening of the acoustic phonon scattering

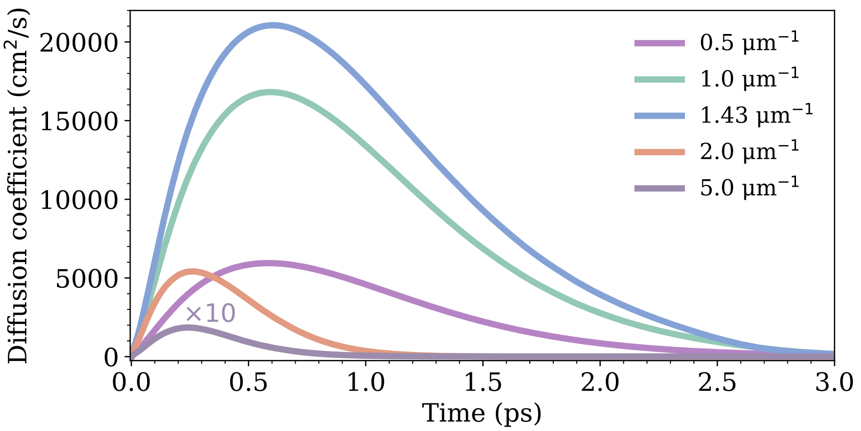

Figure S3: **Momentum-resolved effective diffusion coefficients** at 300 K and $\Delta = +10$ meV. Note that the diffusion coefficient for 5 μm^{-1} (lavender curve) is multiplied by 10 for clarity. The transient superdiffusion is found to increase with group velocity, peaking at 1.43 μm^{-1}, before then dropping significantly for momenta where the acoustic K phonon scattering channel is open, $Q > 1.55$ μm^{-1}.

channel into KK′ excitons (see Fig. 2(c)). In particular, the diffusion coefficient at 5 μm^{-1} is very small compared to the other momenta due to a combination of increased phonon scattering and reduced group velocity.

S.V Momentum-resolved effective polariton velocity

Figure S4 shows the effective velocity of the momentum- and angle-resolved polariton densities, normalized by v_Q^P, for the same five representative momenta as discussed above. Following the same logic, the momentum-resolved results can be divided into two groups based on whether the given momentum falls before or after the opening of the acoustic intervalley scattering pathway between polaritons and KK′ excitons. Velocity components with momenta below the opening of the scattering channel (0.5, 1.0 and 1.43 μm^{-1}) have a slightly increased effective velocity (normalized to v_Q^P) and a much slower decay than those above (2 and 5 μm^{-1}).

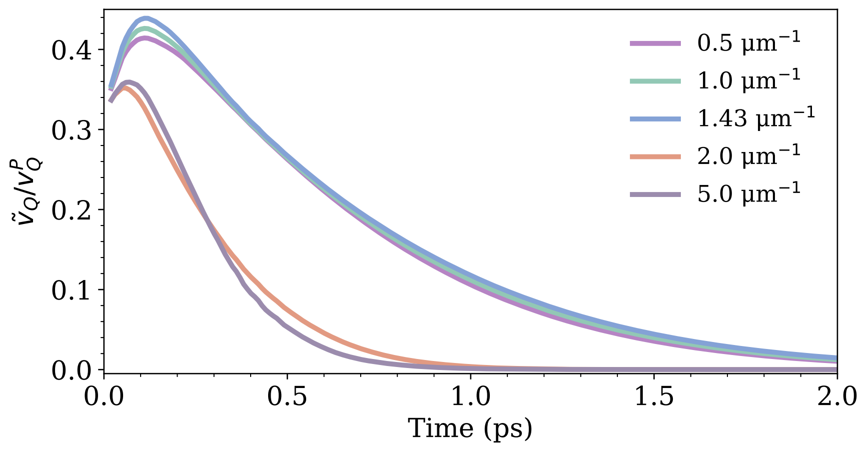

Figure S4: **Momentum- and angle-resolved effective velocity**, divided by v_Q^P, at 300 K and $\Delta = +10$ meV. The effective velocity shows two distinct behaviours depending on the momentum relative to the intervalley scattering opening at $Q = 1.55$ μm^{-1}. Components with momenta below this value peak at a slightly higher fraction of the group velocity and decay more slowly over time.

S.VI Polariton relaxation

To better understand the spatiotemporal dynamics shown in the main text, we discuss here the relaxation in momentum space, resolved at a single spatial point $R = 0$, at $T = 300$ K and $\Delta = +10$ meV. Figure S5(a) shows the time evolution of the integrated occupation over the entire KK and KK′ valleys (including polaritons states in the KK lightcone). Over about 100 ps, there is a rapid transfer of population from the initial hot-exciton distribution in the KK valley to the KK′ valley, driven by efficient scattering via phonon emission. Over the next few hundred femtoseconds, the system thermalizes with the occupation in both valleys smoothly evolving into Boltzmann distributions. This is consistent with previous studies of purely excitonic systems (*57, 65, 77*). Crucially, we find only a weak bottleneck effect; therefore, a significant polariton population can build up in the lightcone on the same timescale as the exciton reservoir thermalizes. This is illustrated in Fig. S5(b), where the increase in the polariton density (calculated by integrating the KK occupation over only the lightcone) is shown. It is this ultrafast feeding of the polariton population that drives the ballistic-like regime observed in the initial expansion period of the polariton cloud (see Figs. 2-4 in the main text).

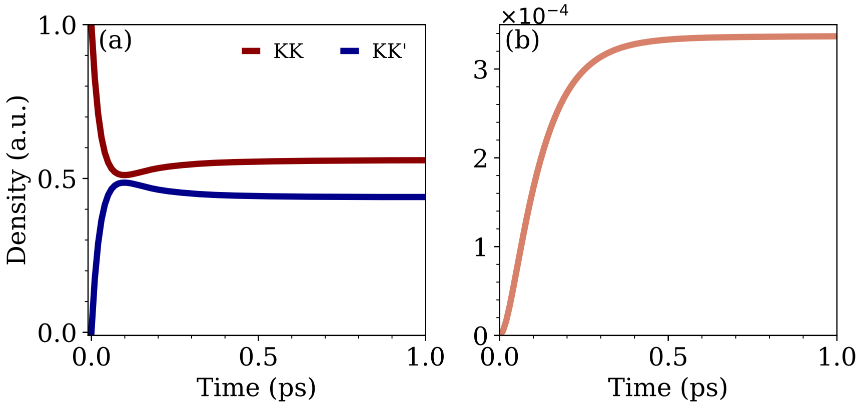

Figure S5: **Relaxation of the integrated polariton occupation** at $R = 0$, $T = 300$ K and $\Delta = +10$ meV. (a) Time evolution of the KK and KK′ density (occupation integrated over all momenta, including both in and outside the lightcone). (b) Time evolution of the polariton density, calculated by integrating the KK occupation over just the lightcone. Note that the densities in this case are on the order of 10^{-4} smaller than in (a).

S.VII Detuning study of cavity-enhanced exciton reservoir broadening

Figure S6 presents a detuning study of the total exciton-polariton broadening at 300 K, i.e., the variance calculated for all states inside and outside the lightcone. Similar to the temperature-dependent study in Fig. 5(a) of the main text, the transient broadening in the first 0.5 ps is approximately the same for all detuning values and the bare monolayer (dashed black line). This indicates a hot-exciton effect unaffected by the presence of a cavity. At steady state, however, we observe a clear detuning dependence of the rate of expansion. As the cavity is blue detuned (Δ becomes more positive), polaritons exhibit increased exciton-like character, consequently weakening the cavity enhancement of the expansion. We confirmed that the enhanced expansion observed in a cavity is characteristic of the entire exciton reservoir, rather than being solely attributable to the rapidly expanding polariton cloud. This was established by an additional study that excluded contributions from exciton polaritons within the lightcone, integrating only states outside it. The results from this closely mirrored those obtained from a full integration.

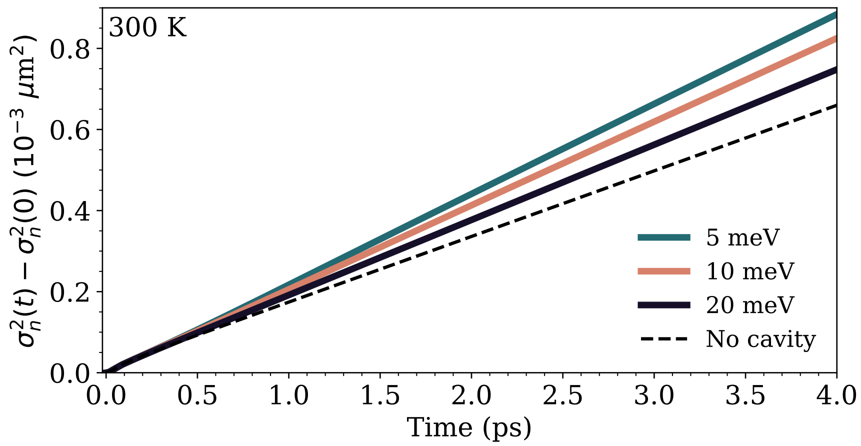

Figure S6: **Detuning study of the cavity-enhanced total exciton reservoir broadening at T = 300 K**. The black dashed line shows the broadening for a bare monolayer with no cavity. At steady state, a clear detuning dependence of the rate of expansion is observed. Exciton polaritons with a more photonic character lead to a stronger cavity enhancement.

S.VIII Dependence on initial excitation conditions

Figure S7 illustrates the impact of the energy of the initial exciton occupation, that is, the centre energy of the Gaussian distribution of excitons in the KK exciton reservoir at $t = 0$. In all preceding results, this was set to 50 meV with a width of 1 meV. By changing this, we can modify the ultrafast thermalization period, i.e., the first few hundred femtoseconds where we observe a ballistic-like expansion of the polariton cloud. In reality, it will also depend on the exact form of non-resonant excitation and the nature of the material's high-energy landscape. Figure S7(a) shows the polariton broadening (integrating over just the lightcone) for a system at 300 K and $\Delta = 10$ meV, examining how it varies for four different initialization energies. We observe that a higher initial KK occupation leads to a slower rate of polariton expansion in real space, indicating a smaller transient effective diffusion. This is attributed to a reduced in-scattering rate from the initial polariton population, which less effectively compensates for the polariton-phonon scattering, resulting in a decreased rate of expansion. This only impacts the transient expansion rate; the spatial width of the polariton cloud in the thermalized limit is found to be approximately the same across all four initialization energies. Interestingly, this dependence on the non-resonant excitation energy is opposite to that observed for bare excitons. Hot excitons with excess kinetic energy diffuse faster than lower-energy

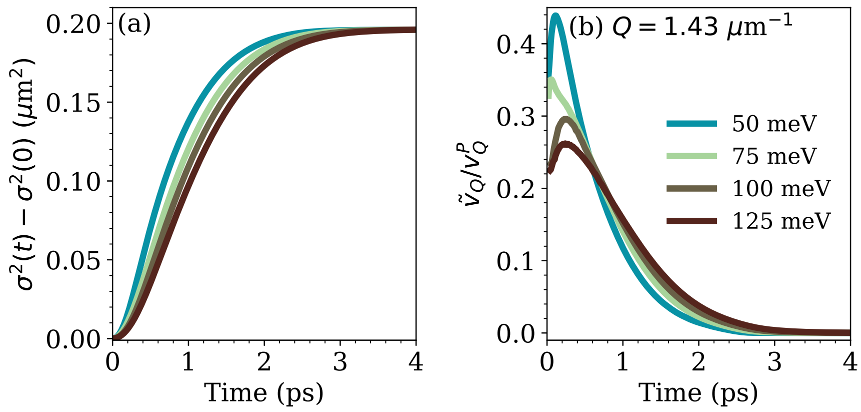

Figure S7: **Dependence on the initial hot-exciton distribution energy.** (a) Polariton broadening shown for different energies of the initial exciton occupation at 300 K and $\Delta = 10$ meV. (b) Corresponding effective velocity of the momentum- and angle-resolved polariton density at $Q = 1.43\ \mu\text{m}^{-1}$.

excitons (*91*), enhancing the transient real-space expansion. For the total broadening (integrating over all states inside and outside the lightcone), we find that the hot-exciton effect dominates the transient expansion, similar to bare excitons.

This behaviour is further confirmed in Fig. S7(b), where the peak effective velocity of the momentum- and angle-resolved polariton density (for $Q = 1.43\ \mu\text{m}^{-1}$) decreases with higher initial occupation energies. However, hotter initial conditions result in a longer temporal tail, causing a larger effective velocity at later times due to the delayed generation of the polariton population. We also find a weak dependence on the initial Gaussian width: a broader initial exciton distribution leads to a lower effective velocity (not shown). Together, these results highlight that the exact relaxation pathway the system takes after non-resonant excitation can significantly impact the type of transient transport behaviour observed in polaritonic systems.

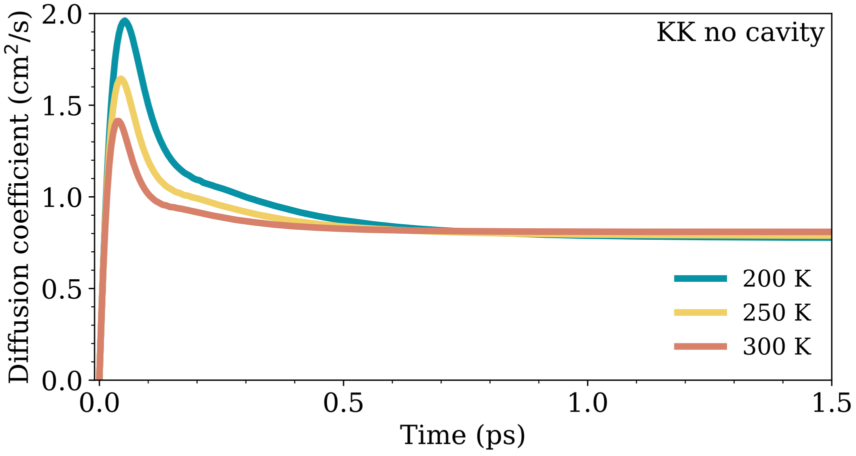

Figure S8: **Temperature dependence of the effective diffusion coefficient** for KK excitons in a bare $MoSe_2$ monolayer. All three temperatures converge to a steady-state value of approximately 0.8 cm^2/s. An equivalent result is found for the diffusion coefficient of KK′ excitons, which limits towards the same value at thermal equilibrium.

S.IX Temperature dependence of bare exciton diffusion in monolayer $MoSe_2$

Figure S8 illustrates the temperature dependence of the KK effective diffusion coefficient for a bare $MoSe_2$ monolayer. Beyond the transient regime, characterized by a peak in diffusion around 100 fs, all three temperatures converge to a steady-state value of approximately 0.8 cm^2/s (*14, 65*). A very similar result is found for the diffusion coefficient of KK′ excitons, which limits towards the same value at thermal equilibrium. At steady state, the temperature insensitivity can be understood from Fick's law as a near-cancellation between competing effects. Specifically, for higher temperatures, the increasing contribution from high-energy exciton occupation via the Boltzmann distribution is offset by the rising exciton-phonon scattering rate.

S.X The polaritonic Fick's law

In the main text we introduced the modified valley/branch-resolved Fick's law for exciton polaritons,

$$\tilde{D}_n^{\mathrm{F}} = \frac{\hbar}{2} \sum_{\mathbf{Q}} \frac{\Gamma_{nQ}}{(\gamma_{nQ} + \Gamma_{nQ})^2} v_{nQ}^2 \frac{f_{nQ}}{\sum_{\mathbf{Q}'} f_{nQ'}}, \tag{S16}$$

where $f_{nQ}/\sum_{\mathbf{Q}'} f_{nQ'}$ is the Boltzmann distribution normalized with respect to the branch/valley n. Equation S16 provides the steady-state diffusion coefficient for the entire exciton polariton population of that branch/valley, including contributions from states both inside and outside the lightcone. It can be derived by linearizing Eq. S13 under the assumption that scattering processes are sufficiently fast that the exciton/polariton occupation only weakly deviates from local quasi-equilibrium (*63*). This means that radiative decay should be weak enough such that it is sensible to talk of a quasi-thermalized system. The additional factor $\Gamma_{nQ}/(\gamma_{nQ} + \Gamma_{nQ})$ in Eq. S16 accounts for depletion of the steady-state occupation due to radiative decay. This is valid when in-scattering into polaritons is dominated from states outside the lightcone (*53*).

The *total* diffusion can be found by summing over all branches and valleys, yielding

$$\begin{aligned}\tilde{D}^{\mathrm{F}} &= \frac{\hbar}{2}\sum_{n}\sum_{\mathbf{Q}}\frac{\Gamma_{nQ}}{(\gamma_{nQ}+\Gamma_{nQ})^2}v_{nQ}^2 f_{nQ}\frac{1}{\sum_{m\mathbf{Q}'} f_{mQ'}} \\ &= \frac{\hbar}{2}\sum_{n}\underbrace{\sum_{\mathbf{Q}}\frac{\Gamma_{nQ}}{(\gamma_{nQ}+\Gamma_{nQ})^2}v_{nQ}^2 \frac{f_{nQ}}{\sum_{\mathbf{Q}''} f_{nQ''}}}_{\tilde{D}_n^{\mathrm{F}}}\underbrace{\frac{\sum_{\mathbf{Q}''} f_{nQ''}}{\sum_{m\mathbf{Q}'} f_{mQ'}}}_{N_n/N} = \sum_n \tilde{D}_n^{\mathrm{F}}\frac{N_n}{N}\end{aligned} \tag{S17}$$

where $N_n = \sum_{\mathbf{Q}} f_{n\mathbf{Q}}$ and $N = \sum_n N_n = \sum_n \sum_{\mathbf{Q}} f_{n\mathbf{Q}}$. Crucially, in the first line we normalized the Boltzmann distribution over all branches and valleys. This reveals that the total steady-state diffusion coefficient is given by the population-weighted sum of valley-resolved diffusion coefficients.

REFERENCES AND NOTES

1. K. F. Mak, C. Lee, J. Hone, J. Shan, T. F. Heinz, Atomically thin MoS_2: A new direct-gap semiconductor. *Phys. Rev. Lett.* **105**, 136805 (2010).

2. G. Wang, A. Chernikov, M. M. Glazov, T. F. Heinz, X. Marie, T. Amand, B. Urbaszek, Colloquium: Excitons in atomically thin transition metal dichalcogenides. *Rev. Mod. Phys.* **90**, 021001 (2018).

3. T. Mueller, E. Malic, Exciton physics and device application of two-dimensional transition metal dichalcogenide semiconductors. *npj 2D Mater. Appl.* **2**, 29 (2018).

4. R. Perea-Causin, D. Erkensten, J. M. Fitzgerald, J. J. P. Thompson, R. Rosati, S. Brem, E. Malic, Exciton optics, dynamics, and transport in atomically thin semiconductors. *APL Mater.* **10**, 100701 (2022).

5. E. Malic, R. Perea-Causin, R. Rosati, D. Erkensten, S. Brem, Exciton transport in atomically thin semiconductors. *Nat. Commun.* **14**, 3430 (2023).

6. D. Jariwala, A. R. Davoyan, J. Wong, H. A. Atwater, Van der Waals materials for atomically-thin photovoltaics: Promise and outlook. *ACS Photonics* **4**, 2962–2970 (2017).

7. J. S. Ross, P. Klement, A. M. Jones, N. J. Ghimire, J. Yan, D. G. Mandrus, T. Taniguchi, K. Watanabe, K. Kitamura, W. Yao, D. H. Cobden, X. Xu, Electrically tunable excitonic light-emitting diodes based on monolayer WSe_2 p–n junctions. *Nat. Nanotechnol.* **9**, 268–272 (2014).

8. J.-L. Brédas, E. H. Sargent, G. D. Scholes, Photovoltaic concepts inspired by coherence effects in photosynthetic systems. *Nat. Mater.* **16**, 35–44 (2017).

9. D. Unuchek, A. Ciarrocchi, A. Avsar, K. Watanabe, T. Taniguchi, A. Kis, Room-temperature electrical control of exciton flux in a van der Waals heterostructure. *Nature* **560**, 340–344 (2018).

10. S. Mouri, Y. Miyauchi, M. Toh, W. Zhao, G. Eda, K. Matsuda, Nonlinear photoluminescence in atomically thin layered WSe_2 arising from diffusion-assisted exciton-exciton annihilation. *Phys. Rev. B* **90**, 155449 (2014).

11. T. Kato, T. Kaneko, Transport dynamics of neutral excitons and trions in monolayer WS_2. *ACS Nano* **10**, 9687–9694 (2016).

12. M. Kulig, J. Zipfel, P. Nagler, S. Blanter, C. Schüller, T. Korn, N. Paradiso, M. M. Glazov, A. Chernikov, Exciton diffusion and halo effects in monolayer semiconductors. *Phys. Rev. Lett.* **120**, 207401 (2018).

13. S. Z. Uddin, H. Kim, M. Lorenzon, M. Yeh, D.-H. Lien, E. S. Barnard, H. Htoon, A. Weber-Bargioni, A. Javey, Neutral exciton diffusion in monolayer MoS_2. *ACS Nano* **14**, 13433–13440 (2020).

14. K. Wagner, J. Zipfel, R. Rosati, E. Wietek, J. D. Ziegler, S. Brem, R. Perea-Causín, T. Taniguchi, K. Watanabe, M. M. Glazov, E. Malic, A. Chernikov, Nonclassical exciton diffusion in monolayer WSe_2. *Phys. Rev. Lett.* **127**, 076801 (2021).

15. C. Carmesin, M. Lorke, M. Florian, D. Erben, A. Schulz, T. O. Wehling, F. Jahnke, Quantum-dot-like states in molybdenum disulfide nanostructures due to the interplay of local surface wrinkling, strain, and dielectric confinement. *Nano Lett.* **19**, 3182–3186 (2019).

16. Z. Li, D. F. Cordovilla Leon, W. Lee, K. Datta, Z. Lyu, J. Hou, T. Taniguchi, K. Watanabe, E. Kioupakis, P. B. Deotare, Dielectric engineering for manipulating exciton transport in semiconductor monolayers. *Nano Lett.* **21**, 8409–8417 (2021).

17. A. Branny, S. Kumar, R. Proux, B. D. Gerardot, Deterministic strain-induced arrays of quantum emitters in a two-dimensional semiconductor. *Nat. Commun.* **8**, 15053 (2017).

18. T. P. Darlington, C. Carmesin, M. Florian, E. Yanev, O. Ajayi, J. Ardelean, D. A. Rhodes, A. Ghiotto, A. Krayev, K. Watanabe, T. Taniguchi, J. W. Kysar, A. N. Pasupathy, J. C. Hone, F. Jahnke, N. J. Borys, P. J. Schuck, Imaging strain-localized excitons in nanoscale bubbles of monolayer WSe_2 at room temperature. *Nat. Nanotechnol.* **15**, 854–860 (2020).

19. E. Malic, M. Selig, M. Feierabend, S. Brem, D. Christiansen, F. Wendler, A. Knorr, G. Berghäuser, Dark excitons in transition metal dichalcogenides. *Phys. Rev. Mater.* **2**, 014002 (2018).

20. J. Zipfel, M. Kulig, R. Perea-Causín, S. Brem, J. D. Ziegler, R. Rosati, T. Taniguchi, K. Watanabe, M. M. Glazov, E. Malic, A. Chernikov, Exciton diffusion in monolayer semiconductors with suppressed disorder. *Phys. Rev. B* **101**, 115430 (2020).

21. R. Rosati, S. Brem, R. Perea-Causín, R. Schmidt, I. Niehues, S. Michaelis de Vasconcellos, R. Bratschitsch, E. Malic, Strain-dependent exciton diffusion in transition metal dichalcogenides. *2D Mater.* **8**, 015030 (2021).

22. A. M. Kumar, D. Yagodkin, R. Rosati, D. J. Bock, C. Schattauer, S. Tobisch, J. Hagel, B. Höfer, J. N. Kirchhof, P. Hernández López, K. Burfeindt, S. Heeg, C. Gahl, F. Libisch, E. Malic, K. I. Bolotin, Strain fingerprinting of exciton valley character in 2D semiconductors. *Nat. Commun.* **15**, 7546 (2024).

23. R. Rosati, R. Schmidt, S. Brem, R. Perea-Causín, I. Niehues, J. Kern, J. A. Preuß, R. Schneider, S. Michaelis de Vasconcellos, R. Bratschitsch, E. Malic, Dark exciton anti-funneling in atomically thin semiconductors. *Nat. Commun.* **12**, 7221 (2021).

24. Z. Sun, A. Ciarrocchi, F. Tagarelli, J. F. Gonzalez Marin, K. Watanabe, T. Taniguchi, A. Kis, Excitonic transport driven by repulsive dipolar interaction in a van der Waals heterostructure. *Nat. Photonics* **16**, 79–85 (2022).

25. F. Tagarelli, E. Lopriore, D. Erkensten, R. Perea-Causín, S. Brem, J. Hagel, Z. Sun, G. Pasquale, K. Watanabe, T. Taniguchi, E. Malic, A. Kis, Electrical control of hybrid exciton transport in a van der Waals heterostructure. *Nat. Photonics* **17**, 615–621 (2023).

26. Z. Li, X. Lu, D. F. Cordovilla Leon, Z. Lyu, H. Xie, J. Hou, Y. Lu, X. Guo, A. Kaczmarek, T. Taniguchi, K. Watanabe, L. Zhao, L. Yang, P. B. Deotare, Interlayer exciton transport in $MoSe_2$/WSe_2 heterostructures. *ACS Nano* **15**, 1539–1547 (2021).

27. J. D. Töpfer, H. Sigurdsson, L. Pickup, P. G. Lagoudakis, Time-delay polaritonics. *Commun. Phys.* **3**, 2 (2020).

28. T. Espinosa-Ortega, T. C. H. Liew, Complete architecture of integrated photonic circuits based on and and not logic gates of exciton polaritons in semiconductor microcavities. *Phys. Rev. B* **87**, 195305 (2013).

29. C. Schneider, M. M. Glazov, T. Korn, S. Höfling, B. Urbaszek, Two-dimensional semiconductors in the regime of strong light-matter coupling. *Nat. Commun.* **9**, 2695 (2018).

30. E. Lopriore, F. Tagarelli, J. M. Fitzgerald, J. F. Gonzalez Marin, K. Watanabe, T. Taniguchi, E. Malic, A. Kis, Enhancing interlayer exciton dynamics by coupling with monolithic cavities via the field-induced Stark effect. *Nat. Nanotechnol.*, 10.1038/s41565-025-01969-2 (2025).

31. B. Han, J. M. Fitzgerald, L. Lackner, R. Rosati, M. Esmann, F. Eilenberger, T. Taniguchi, K. Watanabe, M. Syperek, E. Malic, C. Schneider, Infrared magnetopolaritons in $MoTe_2$ monolayers and bilayers. *Phys. Rev. Lett.* **134**, 076902 (2025).

32. J. K. König, J. M. Fitzgerald, E. Malic, Magneto-optics of anisotropic exciton polaritons in two-dimensional perovskites. *Nano Lett.* **25**, 8519–8526 (2025).

33. M. Wurdack, E. Estrecho, S. Todd, T. Yun, M. Pieczarka, S. K. Earl, J. A. Davis, C. Schneider, A. G. Truscott, E. A. Ostrovskaya, Motional narrowing, ballistic transport, and trapping of room-temperature exciton polaritons in an atomically-thin semiconductor. *Nat. Commun.* **12**, 5366 (2021).

34. T. Freixanet, B. Sermage, A. Tiberj, R. Planel, In-plane propagation of excitonic cavity polaritons. *Phys. Rev. B* **61**, 7233–7236 (2000).

35. M. Steger, G. Liu, B. Nelsen, C. Gautham, D. W. Snoke, R. Balili, L. Pfeiffer, K. West, Long-range ballistic motion and coherent flow of long-lifetime polaritons. *Phys. Rev. B* **88**, 235314 (2013).

36. I. Rosenberg, D. Liran, Y. Mazuz-Harpaz, K. West, L. Pfeiffer, R. Rapaport, Strongly interacting dipolar-polaritons. *Sci. Adv.* **4**, eaat8880 (2018).

37. G. Lerario, D. Ballarini, A. Fieramosca, A. Cannavale, A. Genco, F. Mangione, S. Gambino, L. Dominici, M. de Giorgi, G. Gigli, D. Sanvitto, High-speed flow of interacting organic polaritons. *Light Sci. Appl.* **6**, e16212 (2017).

38. G. G. Rozenman, K. Akulov, A. Golombek, T. Schwartz, Long-range transport of organic exciton-polaritons revealed by ultrafast microscopy. *ACS Photonics* **5**, 105–110 (2018).

39. S. Hou, M. Khatoniar, K. Ding, Y. Qu, A. Napolov, V. M. Menon, S. R. Forrest, Ultralong-range energy transport in a disordered organic semiconductor at room temperature via coherent exciton-polariton propagation. *Adv. Mater.* **32**, e2002127 (2020).

40. M. Balasubrahmaniyam, A. Simkhovich, A. Golombek, G. Sandik, G. Ankonina, T. Schwartz, From enhanced diffusion to ultrafast ballistic motion of hybrid light–matter excitations. *Nat. Mater.* **22**, 338–344 (2023).

41. D. Xu, A. Mandal, J. M. Baxter, S. W. Cheng, I. Lee, H. Su, S. Liu, D. R. Reichman, M. Delor, Ultrafast imaging of polariton propagation and interactions. *Nat. Commun.* **14**, 3881 (2023).

42. R. Su, J. Wang, J. Zhao, J. Xing, W. Zhao, C. Diederichs, T. C. H. Liew, Q. Xiong, Room temperature long-range coherent exciton polariton condensate flow in lead halide perovskites. *Sci. Adv.* **4**, eaau0244 (2018).

43. L. Jin, A. D. Sample, D. Sun, Y. Gao, S. Deng, R. Li, L. Dou, T. W. Odom, L. Huang, Enhanced two-dimensional exciton propagation via strong light–matter coupling with surface lattice plasmons. *ACS Photonics* **10**, 1983–1991 (2023).

44. M. Black, M. Asadi, P. Darman, S. Seçkin, F. Schillmöller, T. A. F. König, S. Darbari, N. Talebi, Long-range self-hybridized exciton-polaritons in two-dimensional ruddlesden–popper perovskites. *ACS Photonics* **11**, 4065–4075 (2024).

45. N. H. M. Dang, S. Zanotti, E. Drouard, C. Chevalier, G. Trippé-Allard, E. Deleporte, C. Seassal, D. Gerace, H. S. Nguyen, Long-range ballistic propagation of 80% excitonic fraction polaritons in a perovskite metasurface at room temperature. *Nano Lett.* **24**, 11839–11846 (2024).

46. H. Shan, L. Lackner, B. Han, E. Sedov, C. Rupprecht, H. Knopf, F. Eilenberger, J. Beierlein, N. Kunte, M. Esmann, K. Yumigeta, K. Watanabe, T. Taniguchi, S. Klembt, S. Höfling, A. V. Kavokin, S. Tongay, C. Schneider, C. Antón-Solanas, Spatial coherence of room-temperature monolayer WSe_2 exciton-polaritons in a trap. *Nat. Commun.* **12**, 6406 (2021).

47. Q. Guo, B. Wu, R. Du, J. Ji, K. Wu, Y. Li, Z. Shi, S. Zhang, H. Xu, Boosting exciton transport in WSe_2 by engineering its photonic substrate. *ACS Photonics* **9**, 2817–2824 (2022).

48. B. Liu, J. Lynch, H. Zhao, B. R. Conran, C. McAleese, D. Jariwala, S. R. Forrest, Long-range propagation of exciton-polaritons in large-area 2D semiconductor monolayers. *ACS Nano* **17**, 14442–14448 (2023).

49. X. Xie, Q. Li, C. Liu, Y. Liu, C. Lee, K. Sun, H. Deng, 2D material exciton-polariton transport on 2D photonic crystals. *Sci. Adv.* **11**, eads0231 (2025).

50. A. N. Osipov, I. V. Iorsh, A. V. Yulin, I. A. Shelykh, Transport regimes for exciton polaritons in disordered microcavities. *Phys. Rev. B* **108**, 104202 (2023).

51. B. Ferreira, R. Rosati, J. M. Fitzgerald, E. Malic, Signatures of dark excitons in exciton–polariton optics of transition metal dichalcogenides. *2D Mater.* **10**, 015012 (2023).

52. B. Ferreira, H. Shan, R. Rosati, J. M. Fitzgerald, L. Lackner, B. Han, M. Esmann, P. Hays, G. Leibeling, K. Watanabe, T. Taniguchi, F. Eilenberger, S. Tongay, C. Schneider, E. Malic, Revealing dark exciton signatures in polariton spectra of 2D materials. *ACS Photonics* **11**, 2215–2220 (2024).

53. J. M. Fitzgerald, R. Rosati, B. Ferreira, H. Shan, C. Schneider, E. Malic, Circumventing the polariton bottleneck via dark excitons in 2D semiconductors. *Optica* **11**, 1346–1351 (2024).

54. F. Tassone, C. Piermarocchi, V. Savona, A. Quattropani, P. Schwendimann, Bottleneck effects in the relaxation and photoluminescence of microcavity polaritons. *Phys. Rev. B* **56**, 7554–7563 (1997).

55. D. Aristov, S. Baryshev, J. D. Töpfer, H. Sigurdsson, P. G. Lagoudakis, Directional planar antennae in polariton condensates. *Appl. Phys. Lett.* **123**, 121101 (2023).

56. G. Berghäuser, E. Malic, Analytical approach to excitonic properties of MoS_2. *Phys. Rev. B* **89**, 125309 (2014).

57. M. Selig, G. Berghäuser, M. Richter, R. Bratschitsch, A. Knorr, E. Malic, Dark and bright exciton formation, thermalization, and photoluminescence in monolayer transition metal dichalcogenides. *2D Mater.* **5**, 035017 (2018).

58. O. A. Ajayi, J. V. Ardelean, G. D. Shepard, J. Wang, A. Antony, T. Taniguchi, K. Watanabe, T. F. Heinz, S. Strauf, X.-Y. Zhu, J. C. Hone, Approaching the intrinsic photoluminescence linewidth in transition metal dichalcogenide monolayers. *2D Mater.* **4**, 031011 (2017).

59. J. M. Fitzgerald, J. J. P. Thompson, E. Malic, Twist angle tuning of Moiré exciton polaritons in van der Waals heterostructures. *Nano Lett.* **22**, 4468–4474 (2022).

60. B. Ferreira, R. Rosati, E. Malic, Microscopic modeling of exciton-polariton diffusion coefficients in atomically thin semiconductors. *Phys. Rev. Mater.* **6**, 034008 (2022).

61. Z. Jin, X. Li, J. T. Mullen, K. W. Kim, Intrinsic transport properties of electrons and holes in monolayer transition-metal dichalcogenides. *Phys. Rev. B* **90**, 045422 (2014).

62. S. Bae, K. Matsumoto, H. Raebiger, K.-i. Shudo, Y.-H. Kim, Ø. S. Handegård, T. Nagao, M. Kitajima, Y. Sakai, X. Zhang, R. Vajtai, P. Ajayan, J. Kono, J. Takeda, I. Katayama, K-point longitudinal acoustic phonons are responsible for ultrafast intervalley scattering in monolayer $MoSe_2$. *Nat. Commun.* **13**, 4279 (2022).

63. O. Hess, T. Kuhn, Maxwell-Bloch equations for spatially inhomogeneous semiconductor lasers. I. Theoretical formulation. *Phys. Rev. A* **54**, 3347–3359 (1996).

64. R. Rosati, R. Perea-Causin, S. Brem, E. Malic, Negative effective excitonic diffusion in monolayer transition metal dichalcogenides. *Nanoscale* **12**, 356–363 (2020).

65. R. Rosati, K. Wagner, S. Brem, R. Perea-Causín, J. D. Ziegler, J. Zipfel, T. Taniguchi, K. Watanabe, A. Chernikov, E. Malic, Non-equilibrium diffusion of dark excitons in atomically thin semiconductors. *Nanoscale* **13**, 19966–19972 (2021).

66. E. Najafi, V. Ivanov, A. Zewail, M. Bernardi, Super-diffusion of excited carriers in semiconductors. *Nat. Commun.* **8**, 15177 (2017).

67. Y.-K. Zhou, X.-Z. Li, Q.-N. Zhou, R.-H. Xing, Y. Zhang, B. Bai, H.-H. Fang, H.-B. Sun, Transient superdiffusion of energetic carriers in transition metal dichalcogenides visualized by ultrafast pump-probe microscopy. *Ultrafast Sci.* **2022**, 0002 (2022).

68. G. L. G. Morganti, R. Rosati, G. D. Brinatti Vazquez, S. Varghese, D. Saleta Reig, E. Malic, N. F. van Hulst, K.-J. Tielrooji, Transient ultrafast and negative diffusion of charge carriers in suspended $MoSe_2$ from multilayer to monolayer. *Nat. Commun.* **16**, 5184 (2025).

69. Z. Guo, Y. Wan, M. Yang, J. Snaider, K. Zhu, L. Huang, Long-range hot-carrier transport in hybrid perovskites visualized by ultrafast microscopy. *Science* **356**, 59–62 (2017).

70. E. Wertz, L. Ferrier, D. D. Solnyshkov, R. Johne, D. Sanvitto, A. Lemaître, I. Sagnes, R. Grousson, A. V. Kavokin, P. Senellart, G. Malpuech, J. Bloch, Spontaneous formation and optical manipulation of extended polariton condensates. *Nat. Phys.* **6**, 860–864 (2010).

71. R. Rosati, K. Wagner, S. Brem, R. Perea-Causín, E. Wietek, J. Zipfel, J. D. Ziegler, M. Selig, T. Taniguchi, K. Watanabe, A. Knorr, A. Chernikov, E. Malic, Temporal evolution of low-temperature phonon sidebands in transition metal dichalcogenides. *ACS Photonics* **7**, 2756–2764 (2020).

72. L. Yuan, B. Zheng, J. Kunstmann, T. Brumme, A. B. Kuc, C. Ma, S. Deng, D. Blach, A. Pan, L. Huang, Twist-angle-dependent interlayer exciton diffusion in WS_2–WSe_2 heterobilayers. *Nat. Mater.* **19**, 617–623 (2020).

73. T. Jacqmin, I. Carusotto, I. Sagnes, M. Abbarchi, D. D. Solnyshkov, G. Malpuech, E. Galopin, A. Lemaître, J. Bolch, A. Amo, Direct observation of Dirac cones and a flatband in a honeycomb lattice for polaritons. *Phys. Rev. Lett.* **112**, 116402 (2014).

74. S. W. Lee, J. S. Lee, W. H. Choi, S.-H. Gong, Ultrathin WS_2 polariton waveguide for efficient light guiding. *Adv. Opt. Mater.* **11**, 2300069 (2023).

75. C. Anton-Solanas, M. Waldherr, M. Klaas, H. Suchomel, T. H. Harder, H. Cai, E. Sedov, S. Klembt, A. V. Kavokin, S. Tongay, K. Watanabe, T. Taniguchi, S. Höfling, C. Schneider, Bosonic condensation of exciton–polaritons in an atomically thin crystal. *Nat. Mater.* **20**, 1233–1239 (2021).

76. M. Kira, S. W. Koch, Many-body correlations and excitonic effects in semiconductor spectroscopy. *Prog. Quantum Electron.* **30**, 155–296 (2006).

77. M. Selig, G. Berghauser, A. Raja, P. Nagler, C. Schuller, T. F. Heinz, T. Korn, A. Chernikov, E. Malic, A. Knorr, Excitonic linewidth and coherence lifetime in monolayer transition metal dichalcogenides. *Nat. Commun.* **7**, 13279 (2016).

78. A. Kormányos, G. Burkard, M. Gmitra, J. Fabian, V. Zólyomi, N. D. Drummond, V. Fal'ko, $k\cdot p$ theory for two-dimensional transition metal dichalcogenide semiconductors. *2D Mater.* **2**, 022001 (2015).

79. S. Brem, J. Zipfel, M. Selig, A. Raja, L. Waldecker, J. D. Ziegler, T. Taniguchi, K. Watanabe, A. Chernikov, E. Malic, Intrinsic lifetime of higher excitonic states in tungsten diselenide monolayers. *Nanoscale* **11**, 12381–12387 (2019).

80. A. Laturia, M. L. Van de Put, W. G. Vandenberghe, Dielectric properties of hexagonal boron nitride and transition metal dichalcogenides: From monolayer to bulk. *npj 2D Mater. Appl.* **2**, 6 (2018).

81. H. Haug, S. W. Koch, *Quantum Theory of the Optical and Electronic Properties of Semiconductors* (World Scientific, 2009).

82. C. Koks, M. Van Exter, Microcavity resonance condition, quality factor, and mode volume are determined by different penetration depths. *Opt. Express* **29**, 6879–6889 (2021).

83. G. Panzarini, L. C. Andreani, A. Armitage, D. Baxter, M. S. Skolnick, V. N. Astratov, J. S. Roberts, A. V. Kavokin, M. R. Vladimirova, M. A. Kaliteevski, Cavity-polariton dispersion and polarization splitting in single and coupled semiconductor microcavities. *Phys. Solid State* **41**, 1223–1238 (1999).

84. S. Dufferwiel, S. Schwarz, F. Withers, A. A. P. Trichet, F. Li, M. Sich, O. Del Pozo-Zamudio, C. Clark, A. Nalitov, D. D. Solnyshkov, G. Malpuech, K. S. Novoselov, J. M. Smith, M. S. Skolnick, D. N. Krizhanovskii, A. I. Tartakovskii, Exciton–polaritons in van der Waals heterostructures embedded in tunable microcavities. *Nat. Commun.* **6**, 8579 (2015).

85. F. Lengers, T. Kuhn, D. E. Reiter, Phonon signatures in spectra of exciton polaritons in transition metal dichalcogenides. *Phys. Rev. B* **104**, L241301 (2021).

86. F. Lengers, T. Kuhn, D. E. Reiter, Theory of the absorption line shape in monolayers of transition metal dichalcogenides. *Phys. Rev. B* **101**, 155304 (2020).

87. T. Sohier, M. Calandra, F. Mauri, Two-dimensional Fröhlich interaction in transition-metal dichalcogenide monolayers: Theoretical modeling and first-principles calculations. *Phys. Rev. B* **94**, 085415 (2016).

88. G. Grosso, G. P. Parravicini, *Solid State Physics* (Academic Press, 2013).

89. J. C. Konig-Otto, M. Mittendorff, T. Winzer, F. Kadi, E. Malic, A. Knorr, C. Berger, W. A. de Heer, A. Pashkin, H. Schneider, M. Helm, S. Winnerl, Slow noncollinear coulomb scattering in the vicinity of the dirac point in graphene. *Phys. Rev. Lett.* **117**, 087401 (2016).

90. J. Schilp, T. Kuhn, G. Mahler, Electron-phonon quantum kinetics in pulse-excited semiconductors: Memory and renormalization effects. *Phys. Rev. B* **50**, 5435–5447 (1994).

91. D. F. Cordovilla Leon, Z. Li, S. W. Jang, P. B. Deotare, Hot exciton transport in WSe_2 monolayers. *Phys. Rev. B* **100**, 241401 (2019).
